# *EBF2* variant identified in a patient with atypical partial lipodystrophy causes adipose fibrosis and dysfunction

**DOI:** 10.1172/JCI192737

**Published:** 2026-01-29

**Authors:** Maria C. Foss-Freitas, Donatella Gilio, Lynn Pais, Eric D. Buras, Romil Kaul Verma, Melanie O’Leary, Heidi L. Rehm, Carmen Glaze, Kathryn Russell, Andre Monteiro da Rocha, Adam Neidert, Patrick Seale, Miriam S. Udler, Elif A. Oral, Tae-Hwa Chun

**Affiliations:** 1 Caswell Diabetes Institute and Metabolism, Endocrinology and Diabetes Division, Department of Internal Medicine, University of Michigan Medical School, Ann Arbor, Michigan, USA.; 2 Department of Clinical and Translational Sciences, University of Pisa, Pisa, Italy.; 3 Division of Genetics and Genomics, Boston Children’s Hospital, Boston, Massachusetts, USA.; 4 Broad Institute Center for Mendelian Genomics and Program in Medical and Population Genetics, Broad Institute of MIT and Harvard, Cambridge, Massachusetts, USA.; 5 Frankel Cardiovascular Regeneration Core Laboratory, University of Michigan, Ann Arbor, Michigan, USA.; 6 Institute for Diabetes, Obesity and Metabolism, Department of Cell and Developmental Biology, Perelman School of Medicine at the University of Pennsylvania, Philadelphia, Pennsylvania, USA.; 7 Biointerfaces Institute, The University of Michigan, Ann Arbor, Michigan, USA.

**Keywords:** Genetics, Metabolism, Adipose tissue, Genetic variation, Transcription

## Abstract

Lipodystrophy (LD) syndromes are characterized by loss of adipose tissue (AT), leading to insulin resistance and the development of metabolic syndrome. We identified a heterozygous nonsense variant in early B cell factor 2 (*EBF2*) (Chr8:26033143C>A, NM_022659.4: c.493G>T, p.E165X) in a patient with atypical partial LD (PLD). The EBF family is crucial for the differentiation and function of various mesenchymal tissues. Through in vitro and in vivo disease models, we discovered that this variant limited adipocyte differentiation and hampered AT remodeling. Heterozygous-knockin (*Ebf2^E165X/+^*) mice showed restricted adipogenesis and defective extracellular matrix remodeling during the post-weaning period and high-fat diet–induced (HFD-induced) AT expansion. A HFD caused abnormal adipocyte hypertrophy, decreased the expression of adiponectin and leptin, and led to glucose intolerance in *Ebf2^E165X/+^* mice. Furthermore, key mitochondrial genes involved in fatty acid metabolism and oxidation were downregulated specifically in *Ebf2^E165X/+^* AT. Our results suggest that EBF2 dysfunction caused by this nonsense variant drives disease pathology, establishing a connection between EBF2 disruption and an atypical form of LD.

## Abstract 2

Using data from a unique patient and a model organism, this study identified a genetic defect that disrupts fat tissue development and function.

## Graphical abstract

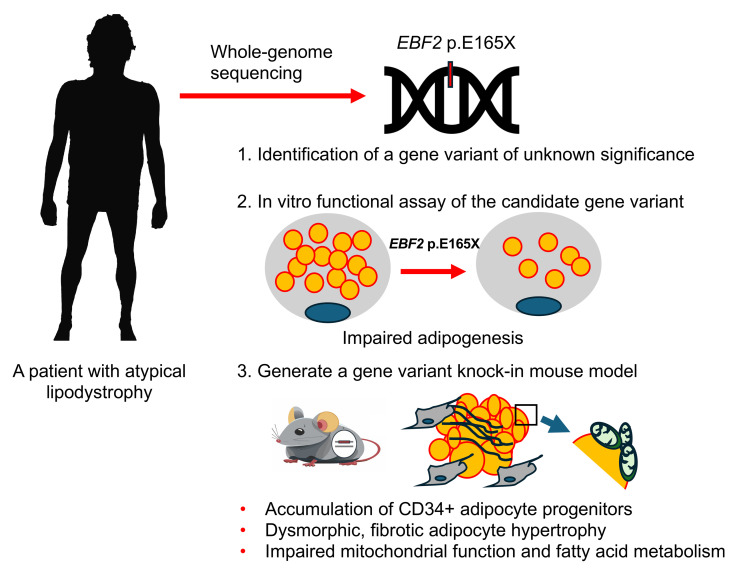

## Introduction

Lipodystrophy (LD) syndromes are characterized by loss of adipose tissue (AT), leading to insulin resistance and metabolic dysfunction (1–3). These diseases present with generalized or partial fat loss (3, 4). The genetic underpinnings of approximately 30% of generalized LD syndromes and 50% of partial lipodystrophy (PLD) syndromes remain unsolved (2). To better define the molecular basis of PLD, we initiated a genetic investigation of affected individuals using whole-genome sequencing. As part of these efforts, we evaluated a young patient with peripheral AT loss who had extensive liver fibrosis, steatohepatitis, nephrosclerosis, insulin resistance, and dyslipidemia (5, 6). Initial genetic screening did not identify causative variants in known LD genes (5). We then performed whole-genome sequencing. By filtering for disruptive gene variants and cross-referencing with clinical databases, we identified a nonsense variant of *EBF2* (8:26033143C>A, c.493G>T, p.E165X).

*EBF2* is a member of the early B cell factor (*EBF*) family, also referred to as Olf-1/EBF (O/E) or Collier/Olf-1/EBF (COE) (7), which plays a crucial role in the differentiation of multiple cell lineages (8–14). The *EBF* family promotes adipocyte differentiation (8), and reduced *EBF1* expression is linked to limited adipocyte hyperplasia, adipocyte hypertrophy, and insulin resistance in humans (15). *Ebf2* regulates mouse brown adipocyte fat in concert with *Pparg* (16). In white adipose tissue (WAT), *Zfp423* antagonizes *Ebf2* to suppress the thermogenic program (17). In addition, *EBF2* is enriched in a subset of human visceral adipocytes and linked to an increased waist-to-hip ratio (18). Moreover, GWASs identified *EBF2* variants associated with hypertension (19), diabetic nephropathy, and visceral adipose mass (20).

On the basis of these findings, we hypothesized that the *EBF2* stop-gain variant (p.E165X) identified in this patient could cause PLD. The truncated variant, EBF2 (1-164), suppressed adipocyte differentiation in 3T3-L1 cells that express endogenous *Ebf1*, *Ebf2*, and *Ebf3* (8), raising the possibility that EBF2 (1-164) acts as a dominant-negative inhibitor of adipogenesis. However, in vitro adipogenesis may differ from in vivo processes, which are influenced by humoral factors (21), the 3D structure of tissues (22), and oxygenation status (23, 24). Further, AT development involves mesenchymal cell proliferation, adipocyte differentiation (25–27), extracellular matrix (ECM) remodeling (22), and angiogenesis (28). In addition, AT development and expansion processes vary across perinatal, postnatal, and adult stages and differ between fat depots (29, 30), with distinct transcriptional networks governing AT development and function (25–27). To investigate the pathological effects of the *EBF2* nonsense variant on AT development, we generated a knockin (KI) mouse model carrying the single-nucleotide change identified in our patient (*Ebf2^E165X/+^*). The *Ebf2^E165X/+^* mice exhibited restricted adipogenesis and impaired ECM remodeling, resulting in AT dysfunction and the persistent presence of a collagen-rich ECM, similar to what we observed in our patient. Using primary mouse preadipocytes, we found that the *EBF2* p.E165X variant caused cell-autonomous defects in adipocyte differentiation and maturation. These findings demonstrate that EBF2 dysfunction disrupts AT development and function.

## Results

### Case presentation.

**Figure 1:**
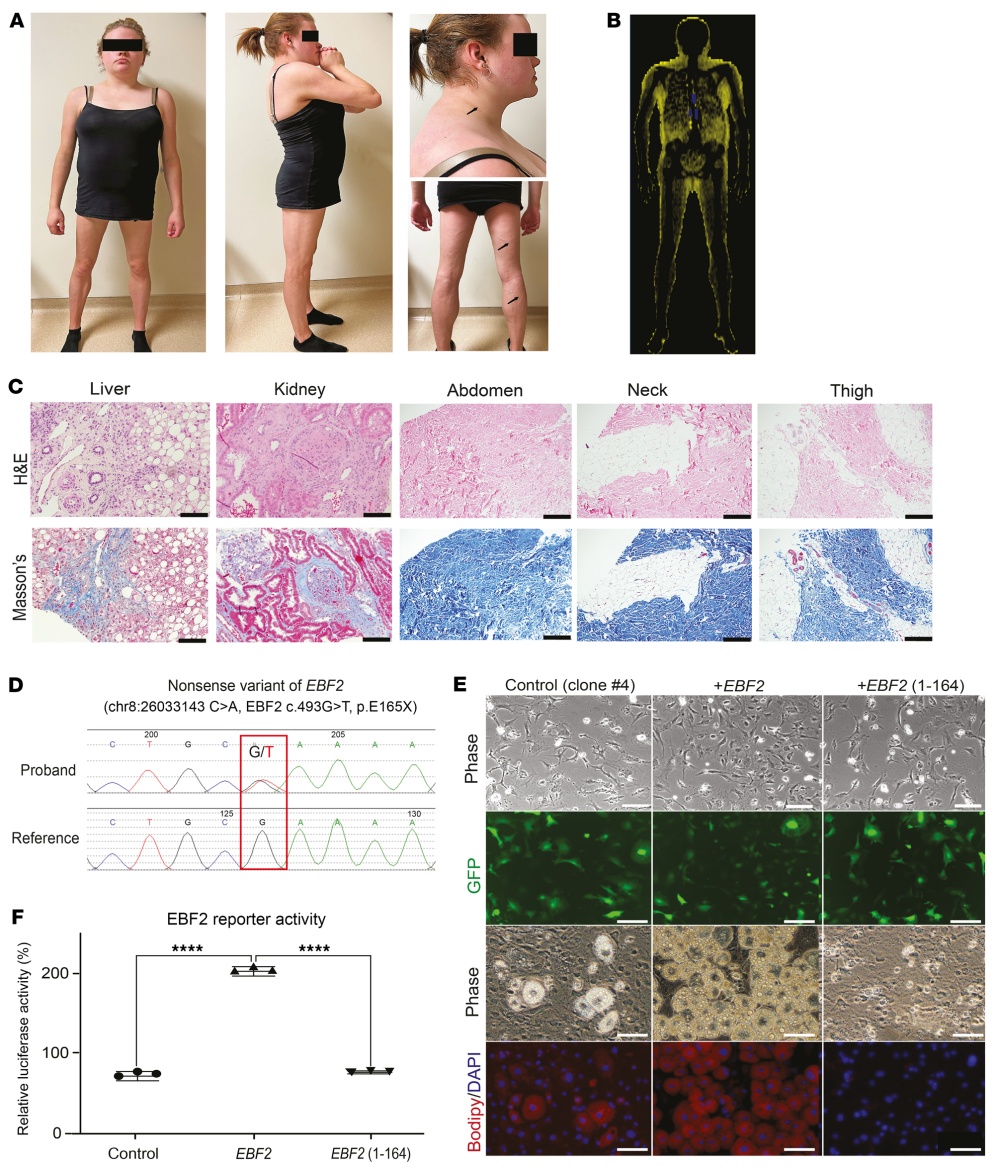
Identification of the *EBF2* p.E165X variant and in vitro functional screening. (**A**) Photographs of a patient with atypical PLD showing altered fat distribution, a dorsal neck fat pad with acanthosis (arrow), and prominent veins in the legs (arrows). (**B**) Whole-body dual-energy X-ray absorptiometry (DEXA) “fat shadow.” (**C**) H&E- and Masson’s trichrome–stained images of liver, kidney, and subcutaneous WAT (abdomen, neck, thigh); collagen is shown in blue. (**D**) Sanger sequencing showing G>T substitution. (**E**) 3T3-L1 cells transduced with control, full-length EBF2, or truncated EBF2 (1-164). GFP (green); BODIPY (red); DAPI (blue). (**F**) Luciferase reporter assay in HEK cells (*n* = 3). Scale bars: 100 μm (**C** and **E**). ****P < 0.0001, by 1-way ANOVA with Tukey’s test (**F**). Data indicate the mean ± SEM. Each dot represents a biological replicate (see also Supplemental Figure 1).

The patient is a 23-year-old woman with PLD (Figure 1, A and B), who first presented for medical attention at age 9 with hypertriglyceridemia and hepatomegaly. She was referred to us at 11 years of age, when AT loss affecting the hips and legs was noted. Although her complex clinical presentation has been previously reported (5, 6), it is worthwhile to review key clinical details. She was born as 1 of a pair of fraternal twins, conceived through in vitro fertilization by her 38-year-old parents. Her father, diagnosed with atypical lupus, type 2 diabetes, and heart failure, died from sudden cardiac arrest on the night of the embryo transfer. She and her fraternal twin were born to her mother at 38 weeks of pregnancy after an uneventful pregnancy, other than the trauma of the father’s unexpected death. The patient was observed to have an umbilical hernia and contracture of the fifth digit of the right hand. She showed no neurodevelopmental delays and achieved all developmental milestones without issues. Additionally, she displayed normal baby fat during her first year of life. Hepatomegaly was detected at age 8.5 years. Labs done in that year demonstrated hypertriglyceridemia and elevated liver enzymes. Her pediatrician also noted scoliosis. The patient was referred to our clinic after a local endocrinologist noted fat loss in her extremities and prominent veins. During the interview, the patient and her mother reported that an unusual fat distribution in the legs was noticed as early as 6 years of age, when she began playing soccer, and her teammates commented on her leg appearance. In our initial evaluation at age 11, the clinical examination revealed signs of PLD, characterized by fat loss in the lower limbs, with palpable AT in the abdomen and increased fat deposits around the face, neck, and upper trunk. The liver was enlarged, with a total liver span of 17 cm, and the abdomen was protuberant. She had no palpable breast tissue and no signs of puberty. No LD phenotype was observed in her fraternal twin brother, older sister, or mother. Labs demonstrated hypertriglyceridemia, insulin resistance, and prediabetes. Additional lab findings included low-titer, anti-glutamic acid decarboxylase (GAD) antibodies, low complement C4 levels, elevated liver transaminases, and massive proteinuria. In the next decade, she developed primary hypogonadotropic hypogonadism, clinically complicated diabetes, and hypertriglyceridemia (5, 6). Also, progressive elevations in liver enzymes and worsening proteinuria were observed, prompting liver and kidney biopsies. Histological examination of the liver showed hepatocyte ballooning with excess lipid accumulation and fibrosis (Figure 1C); the kidneys showed fibrotic ECM accumulation in the glomerulus and interstitium, described by the case pathologist as “Alport-like pathology” (Figure 1C). Biopsies of subcutaneous AT depots (abdomen, neck, and thigh) showed abnormal architecture, with accumulated ECM replacing adipocytes (Figure 1C).

### Molecular investigations identified a nonsense variant in EBF2 (p.E165X) as potentially pathogenic.

We attempted to uncover the molecular basis of the presentation in our patient. First, a LD gene panel (*AGPAT2, AKT2, BSCL2, CAV1, CIDEC, LMNA, PLIN1, PPARG, PTRF, TBC1D4,* and *ZMPSTE24*) (5) detected no known or unknown variants. Subsequently, we performed whole-exome sequencing (WES) with blood samples from the proband, the mother, and the 2 siblings. A detailed list of unique variants is available in Supplemental Table 1. Two protein-truncating rare variants (*LEKR1* p.G123X and *EBF2* p.E165X) were among the heterozygous variants not shared by her family members. Our research laboratory reported these 2 variants as genetic variants of unknown significance. To gather confirmatory evidence and ensure that no other genomic findings explained the patient’s clinical phenotype, we contacted the Broad Institute to undertake whole-genome analyses of the proband and her mother. The variant call set was uploaded to seqr (31) for collaborative study, and then the Broad Institute Center for Mendelian Genomics (CMG) performed variant filtration searches (31) based on the suspected mode of inheritance, the pathogenicity reported in ClinVar, the type of variant, the frequency in population databases, and the variant call quality. We assessed candidate variants for genotypic and phenotypic concordance with known disease genes using Online Mendelian Inheritance in Man (OMIM) and a targeted literature review. As no candidates were identified in known disease genes, we screened high-impact variants in genes of uncertain significance (GUS). Among them, we identified a stop-gain variant in *EBF2*, which was also noted in our prior WES (Supplemental Figure 1A; supplemental material available online with this article; https://doi.org/10.1172/JCI192737DS1). This *EBF2* variant was confirmed with targeted Sanger sequencing (Figure 1D). The heterozygous nonsense variant of *EBF2* (NM_022659.4) was found in exon 6, leading to the premature termination of EBF2 at amino acid position 165 (chr8:26033143 C>A, *EBF2* c.493G>T, p.E165X). Genomic DNA was then isolated from the patient’s father’s hair collected from his hairbrush. Whole-genome sequencing demonstrated the identical *EBF2* variant (chr8:26033143 C>A), thereby confirming paternal inheritance (Supplemental Figure 1B).

There is substantial evidence linking *EBF2* to a LD-like phenotype. At the gene level, the Human Genetic Evidence “HuGE” Calculator indicated “very strong” support for associations between *EBF2* and type 2 diabetes, body fat distribution (waist/hip ratio, visceral/gluteofemoral adipose ratio, visceral/abdominal adipose ratio, visceral AT adjusted for BMI), and cardiometabolic phenotypes (hypertension, systolic blood pressure, diastolic blood pressure, pulse pressure, HDL cholesterol, triglycerides) (Common Metabolic Diseases Knowledge Portal [CMDKP]: hugeamp.org). In large-scale GWASs, the G allele of *EBF2* SNP (rs17818197) is associated with an increased risk of type 2 diabetes (OR 1.02, *P* = 7.69 × 10^–12^) (31); reduced HDL cholesterol (β –0.0058, *P* = 1.96 × 10^–6^) (32); increased systolic blood pressure (β 0.0082, *P* = 2.8 × 10^-6^) (HuGEAMP); an increased risk of hypertension (OR 1.02, *P* = 4.5 × 10^–7^) (33); and increased triglyceride levels (β 0.0061, *P* = 1.67 × 10^–5^) (32). This phenotypic pattern aligns with a LD-like clinical presentation (34, 35). Additionally, rare variant burden testing for *EBF2* in 344,692 individuals identified an association of 198 predicted deleterious missense and loss-of-function (LoF) variants in *EBF2* with increased random glucose levels (*P* = 0.0028) (36).

*EBF2* is highly constrained for protein-truncating variation in the general population, with a probability of LoF intolerance (pLI) score of 1.0 and an observed/expected (o/e) LoF ratio of 0.26 (37, 38). We manually reviewed the LoF variants in gnomAD (Supplemental Table 2) using the advanced variant classification framework (37). Of 57 predicted LoF (pLoF) variants, 25 (44%) are not expected to result in protein truncation for a variety of reasons, including in-frame splicing (*n* = 7), termination in the last exon or last 50 bp of the penultimate exon (*n* = 8), genotyping error (*n* = 1), and presence in a nonbiologically relevant transcript (*n* = 9). Of the remaining 32 variants predicted to result in LoF, 5 were present in gnomAD version 3.1.2. As gnomAD version 4 includes samples from biobanks and disease-specific studies, and version 3.1.2 has controls from cardiovascular and diabetes cohorts, some individuals may display symptoms of LD. On the basis of these findings, we considered the *EBF2* stop-gain variant identified in this patient to be potentially causative of the patient’s PLD-related metabolic phenotype.

### EBF2 p.E165X variant impairs adipogenesis in vitro.

We have utilized 3T3-L1 adipocyte differentiation for the initial functional screening of candidate gene variants identified in our patients with LD syndromes. *Ebf2* knockdown in 3T3-L1 preadipocytes using 2 independent siRNA oligonucleotides (Supplemental Figure 2, A–C, and Supplemental Table 3) reduced lipid accumulation and decreased the expression of the adipocyte genes *Pparg* and *Fabp4* (Supplemental Figure 2, A and C). To assess the activity of the identified *EBF2* variant, we used lentiviral vectors to express full-length and truncated variant (1-164) forms of *EBF2* in 3T3-L1 preadipocytes following silencing of endogenous mouse *Ebf2*. We used 5 different lentiviral shRNA constructs to stably knock down *Ebf2* (Supplemental Table 4) and chose a clone (clone 4) that showed specific suppression of *Ebf2* relative to *Ebf1*. As expected, these cells showed low levels of adipocyte differentiation (Figure 1E). Full-length *EBF2* restored robust adipogenesis (Figure 1E), whereas the variant *EBF2* (1-164) did not (Figure 1E), indicating LoF for the *EBF2* (1-164) variant. Consistent with this, the truncated EBF2 variant failed to activate transcription from an EBF2-driven reporter gene (Figure 1F). Together, these results suggest that the truncated EBF2 variant is a LoF mutation.

### The EBF2 p.E165X variant impairs postnatal AT expansion and remodeling in vivo.

Given the in vitro results, we hypothesized that the truncated *EBF2* variant may have caused AT loss in our patient. To test this hypothesis in vivo, we generated a KI mouse model harboring the observed nonsense variant (*Ebf2^E165X/+^*) using CRISPR/Cas9 genome editing (39). Although we had no difficulty obtaining heterozygous KI mice, we rarely obtained surviving homozygous mice (*Ebf2^E165X/E165X^*) on the C57BL/6J background. However, a few *Ebf2^E165X/E165X^* mice on a mixed genetic background were successfully weaned. A surviving female *Ebf2^E165X/E165X^* mouse lacked perigonadal WAT and showed rudimentary subcutaneous and inguinal WAT with dysmorphic adipocytes and extensive accumulation of eosinophilic fibrillar structures (Supplemental Figure 2D). The WAT showed extensive fibrosis, characterized by a limited number of adipocytes surrounded by excess ECM proteins (Supplemental Figure 2, D–F). Unlike homozygous KI mice, *Ebf2^E165X/+^* mice were born without noticeable defects, and there was no difference in weight between *Ebf2*^+/+^ and *Ebf2^E165X/+^* mice at 8 weeks of age (Supplemental Figure 3A). *Ebf2*^+/+^ and *Ebf2^E165X/+^* mice showed comparable weight gains (Supplemental Figure 3B) and food intake (Supplemental Figure 3C) during an additional 8 weeks of chow diet (CD) feeding. Moreover, *Ebf2^+/+^* and *Ebf2^E165X/+^* mice had similar body compositions as assessed by nuclear magnetic resonance–based (NMR-based) imaging, the percentage of inguinal WAT (IWAT), gonadal WAT (GWAT), and brown adipose tissue (BAT) mass, and liver weights (Supplemental Figure 3, D–F). Fasting serum glucose levels were not different between the groups (Supplemental Figure 3G), but insulin levels were higher in male *Ebf2^E165X/+^* mice than in male *Ebf2^+/+^* mice (Supplemental Figure 3H). Plasma glucose levels during the intraperitoneal glucose tolerance test (IPGTT) were similar between the groups (Supplemental Figure 3I); however, fasting serum triglyceride levels were elevated in male *Ebf2^E165X/+^* mice (Supplemental Figure 3J). The livers showed no histological differences between the groups (Supplemental Figure 3K).

**Figure 2:**
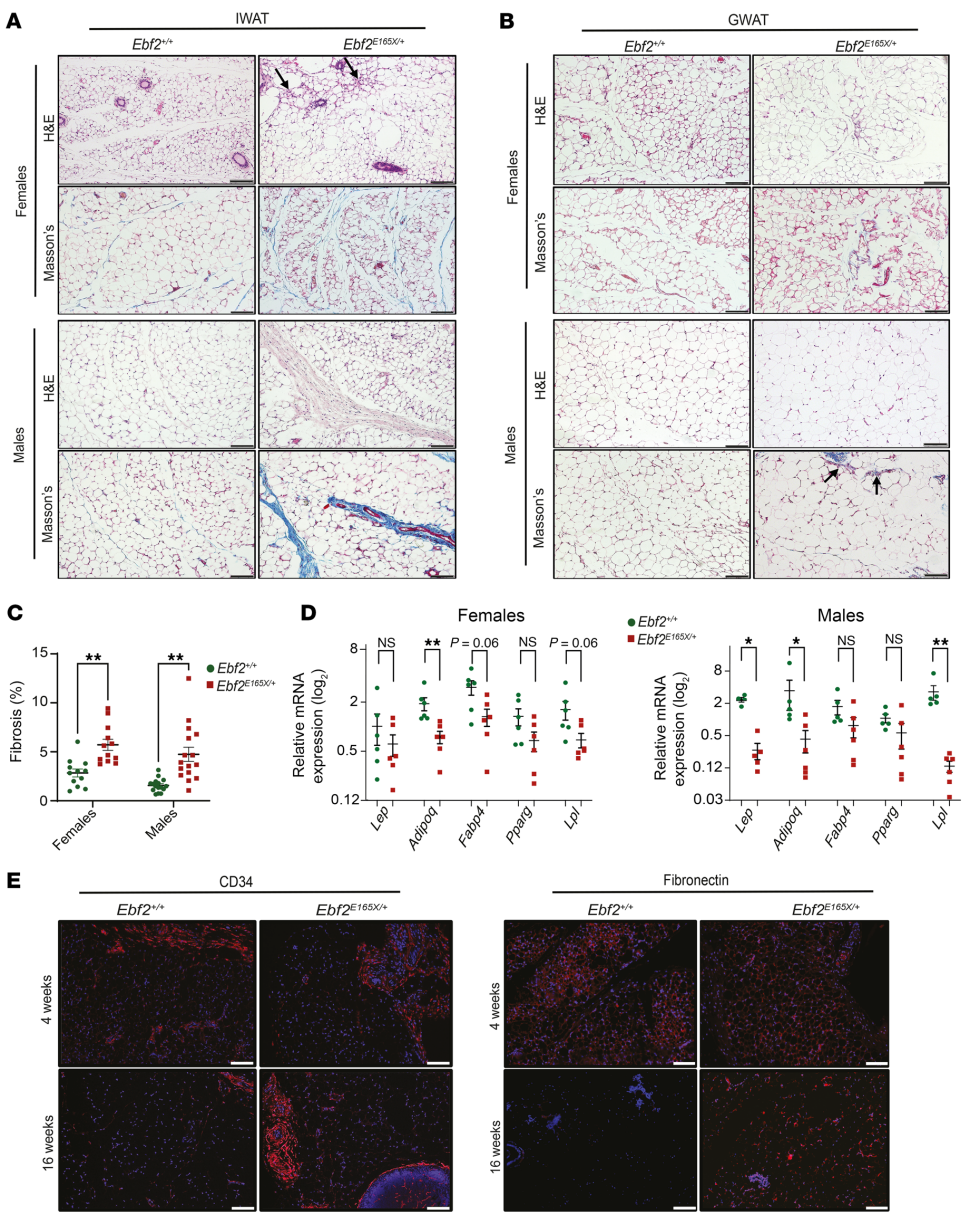
*EBF2* p.E165X impairs postnatal AT expansion and remodeling. (**A** and **B**) H&E- and Masson’s trichrome–stained images of IWAT (**A**) and GWAT (**B**) from 16-week-old mice (*n* = 4). Collagen (blue); stromal clusters (arrows). (**C**) Quantification of IWAT fibrosis (percentage) (*n* = 3–4). (**D**) RT-qPCR analysis of adipogenic genes in IWAT (*n* = 4–6). (**E**) IWAT immunofluorescence images of CD34 and fibronectin (red); DAPI (blue). Scale bars: 100 μm (**A**, **B**, and **E**). *P < 0.05, **P < 0.01 by 2-way ANOVA with Tukey’s test (**C**) and 2-tailed, unpaired Student’s t test (**D**). and 2-way ANOVA with Tukey’s test (**C**). Data indicate the mean ± SEM. Each dot represents 1 mouse (**D**) or a high-powered field (HPF) (**C**). See also Supplemental Figures 2 and 3.

We next evaluated the histological features of WAT from *Ebf2^+/+^* and *Ebf2^E165X/+^* mice. H&E staining of IWAT revealed the heterogeneity of adipocyte size in *Ebf2^E165X/+^* mice (Figure 2A). Notably, smaller adipocytes were typically surrounded by clusters of stromal cells (Figure 2A). *Ebf2^E165X/+^* IWAT also displayed groups of adipocytes surrounded by thick collagen fibrils stained blue with Masson’s trichrome (MT) staining (Figure 2A). In GWAT, we observed less heterogeneity in adipocyte size (Figure 2B). However, the stromal cell clusters were found preferentially in *Ebf2^E165X/+^* mice (Figure 2B) and were often adjacent to collagenous materials stained blue with MT (Figure 2B). Quantification of IWAT fibrosis based on the MT-positive area showed increased fibrosis in tissues from *Ebf2^E165X/+^* mice relative to those from *Ebf2^+/+^* controls (Figure 2C). Given the alterations in AT structure, we also analyzed the expression of key adipocyte genes in WAT depots of *Ebf2^+/+^* and *Ebf2^E165X/+^* mice. *Adipoq* and *Lpl* mRNA expression was reduced in IWAT (Figure 2D). *Lep* expression was also decreased in male *Ebf2^E165X/+^* mice (Figure 2D). Mice of the 2 genotypes showed equivalent expression of these adipocyte genes in GWAT (Supplemental Figure 3L), suggesting that IWAT structure and function were selectively affected in *Ebf2^E165X/+^* mice.

To further investigate when the differences in IWAT structure between *Ebf2*^+/+^ and *Ebf2^E165X/+^* mice manifest, we analyzed IWAT of mice at 4 and 16 weeks of age. Notably, at 4 weeks, we observed no substantial difference in IWAT structure between *Ebf2*^+/+^ and *Ebf2*^E165X/+^ mice. In both groups of mice, IWAT had adipocytes of varying sizes and discrete regions of intense eosin staining (Supplemental Figure 4A) and closer examination revealed clusters of smaller adipocytes (Supplemental Figure 4A). MT staining showed the persistent presence of thick collagen fibrils and the reminiscence of the primordial mesenchyme (Supplemental Figure 4B). Despite the accumulation of collagenous materials in *Ebf2^E165X/+^* mice, *Col1a1* transcript levels were downregulated, indicating that collagen persistence probably reflects impaired ECM remodeling rather than increased synthesis (Supplemental Figure 4C). Immunofluorescence staining revealed the presence of fibronectin and type I and VI collagens adjacent to CD34^+^ cells (Figure 2E and Supplemental Figure 4, D, F, and G). IWAT from 16-week-old *Ebf2*^+/+^ mice displayed a reduction in ECM proteins (collagens and fibronectin), as well as a decrease in the number of CD34^+^ cells, compared with the 4-week-old *Ebf2*^+/+^ mice (Figure 2E and Supplemental Figure 4, D–I). By contrast, IWAT from 16-week-old *Ebf2*^E165X/+^ mice, both male and female, had excess ECM deposits enmeshed with CD34^+^ cells (Figure 2E and Supplemental Figure 4, D and E). The persistence of CD34^+^ cells surrounded by ECM deposits in *Ebf2^E165X/+^* mice suggests a critical role for *Ebf2* in coordinating ECM remodeling and adipocyte maturation, biological processes essential for AT expansion in mice between 4 and 16 weeks of age.

### The EBF2 p.E165X variant disrupts high-fat diet–induced AT remodeling and expansion.

**Figure 3:**
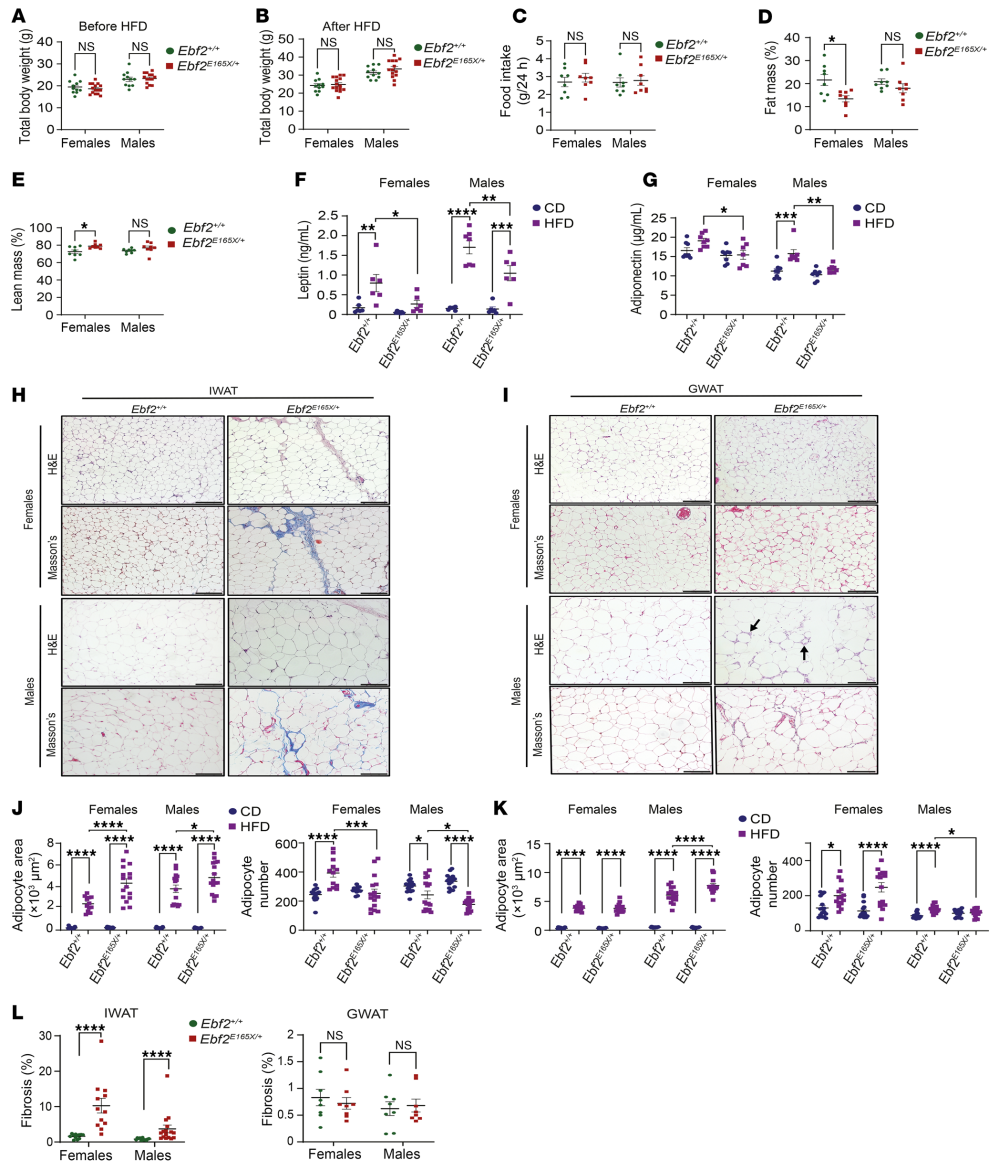
*EBF2* p.E165X disrupts HFD-induced adipose remodeling. Eight-week-old mice were fed a HFD for 2 months (*n* = 11–15 per group). (**A** and **B**) Body weight at baseline and after HFD feeding. (**C**) Food intake at week 8 (*n* = 8/group). (**D** and **E**) Fat and lean mass by NMR (*n* = 8/group). (**F** and **G**) Serum leptin and adiponectin levels (*n* = 6–8/group). (**H** and **I**) H&E- and Masson’s trichrome–stained images of IWAT and GWAT showing collagen (blue) and immune infiltrates (arrows). Scale bars: 100 μm. (**J** and **K**) Quantification of adipocyte area and numbers in IWAT (**J**) and GWAT (**K**) (*n* = 3–4/group). (**L**) IWAT and GWAT fibrosis (percentage) (*n* = 3–4/group). *P < 0.05, **P < 0.01, ***P < 0.001, and ****P < 0.0001, by 2-way ANOVA with Tukey’s test (**A**–**G** and **J**–**L**). Data indicate the mean ± SEM. Each dot represents 1 mouse (**A**–**G**) or a HPF (**J**–**L**). See also Supplemental Figures 4–6.

To determine whether diet interacts with the presence of the *Ebf2* variant, we challenged a group of *Ebf2^+/+^* and *Ebf2^E165X/+^* mice with a 45% kcal high-fat diet (HFD) for 2 months, starting at 8 weeks of age. A few months of HFD feeding is sufficient to induce AT remodeling (30–33). *Ebf2^+/+^* and *Ebf2^E165X/+^* mice, both male and female, started at the same weight (Figure 3A) and gained comparable amounts of weight after 8 weeks of HFD feeding (Figure 3B) with similar food intake (Figure 3C). Although *Ebf2^+/+^* and *Ebf2^E165X/+^* mice gained the same amount of weight, NMR-based body composition analysis showed a reduced percentage of fat (lipid) mass and a higher percentage of lean mass in female *Ebf2^E165X/+^* mice compared with *Ebf2*^+/+^ mice (Figure 3, D and E). A HFD increased serum leptin levels in both *Ebf2^+/+^* and *Ebf2^E165X/+^* mice, but the response was attenuated in *Ebf2^E165X/+^* mice (Figure 3F). Serum adiponectin levels were lower in *Ebf2^E165X/+^* mice than in *Ebf2^+/+^* mice (Figure 3G). However, we detected no differences in IWAT or GWAT mass between *Ebf2*^+/+^ and *Ebf2^E165X/+^* mice in either sex, suggesting the shift of lipid to nonlipid constituents in female *Ebf2^E165X/+^* IWAT (Supplemental Figure 5A). Indeed, histological analysis revealed increased stromal cell clusters and collagen fiber accumulation in *Ebf2^E165X/+^* IWAT (Figure 3H). Additionally, GWAT from *Ebf2^E165X/+^* mice showed more infiltration of immune cells and crown-like structures than did GWAT from *Ebf2*^+/+^ mice (Figure 3I), particularly in males, suggesting an enhanced inflammatory response in *Ebf2^E165X/+^* mice. The adipocyte area in IWAT increased in both *Ebf2*^+/+^ and *Ebf2*^E165X/+^ mice following HFD feeding, with hypertrophy more pronounced in *Ebf2*^E165X/+^ mice (Figure 3J). Adipocyte numbers were correspondingly lower in IWAT of *Ebf2^E165X/+^* compared with *Ebf2*^+/+^ mice (Figure 3J). GWAT also showed augmented HFD-dependent adipocyte hypertrophy with decreased adipocyte numbers in *Ebf2^E165X/+^* male mice relative to *Ebf2^+/+^* male mice (Figure 3K).The quantified fibrosis index was higher in *Ebf2^E165X/+^* IWAT, but not in GWAT (Figure 3L). These findings indicate that a HFD promotes adipocyte hypertrophy while limiting hyperplasia in *Ebf2*^E165X/+^ mice, a pattern reminiscent of the restricted adipogenesis with paradoxical hypertrophy reported in *Ebf1*-null mice (15).

At the gene expression level, *Adipoq* was decreased in IWAT and GWAT of male *Ebf2^E165X/+^* mice compared with *Ebf2^+/+^* mice (Supplemental Figure 5B). *Lpl* was also lower in IWAT of male *Ebf2^E165X/+^* mice, while no differences were observed in other adipocyte genes, such as *Pparg*, *Fabp4*, and *Lep*, when the mice were fed a HFD (Supplemental Figure 5B). We detected no gene expression changes in WAT depots between female *Ebf2^+/+^* and *Ebf2^E165X/+^* mice fed a HFD (Supplemental Figure 5C). A HFD did not alter the signals for type I and VI collagens in IWAT of mice compared with mice in the CD group in either genotype. However, *Ebf2^E165X/+^* mice continued to exhibit persistent deposition of these collagens compared with *Ebf2^+/+^* mice (Supplemental Figure 5D). In GWAT, the signals for type I and VI collagens remained unchanged following HFD feeding, although the type I collagen signal tended to be higher in female *Ebf2^E165X/+^* mice (Supplemental Figure 5E).

### EBF2 nonsense variant worsens HFD-associated metabolic phenotypes.

**Figure 4:**
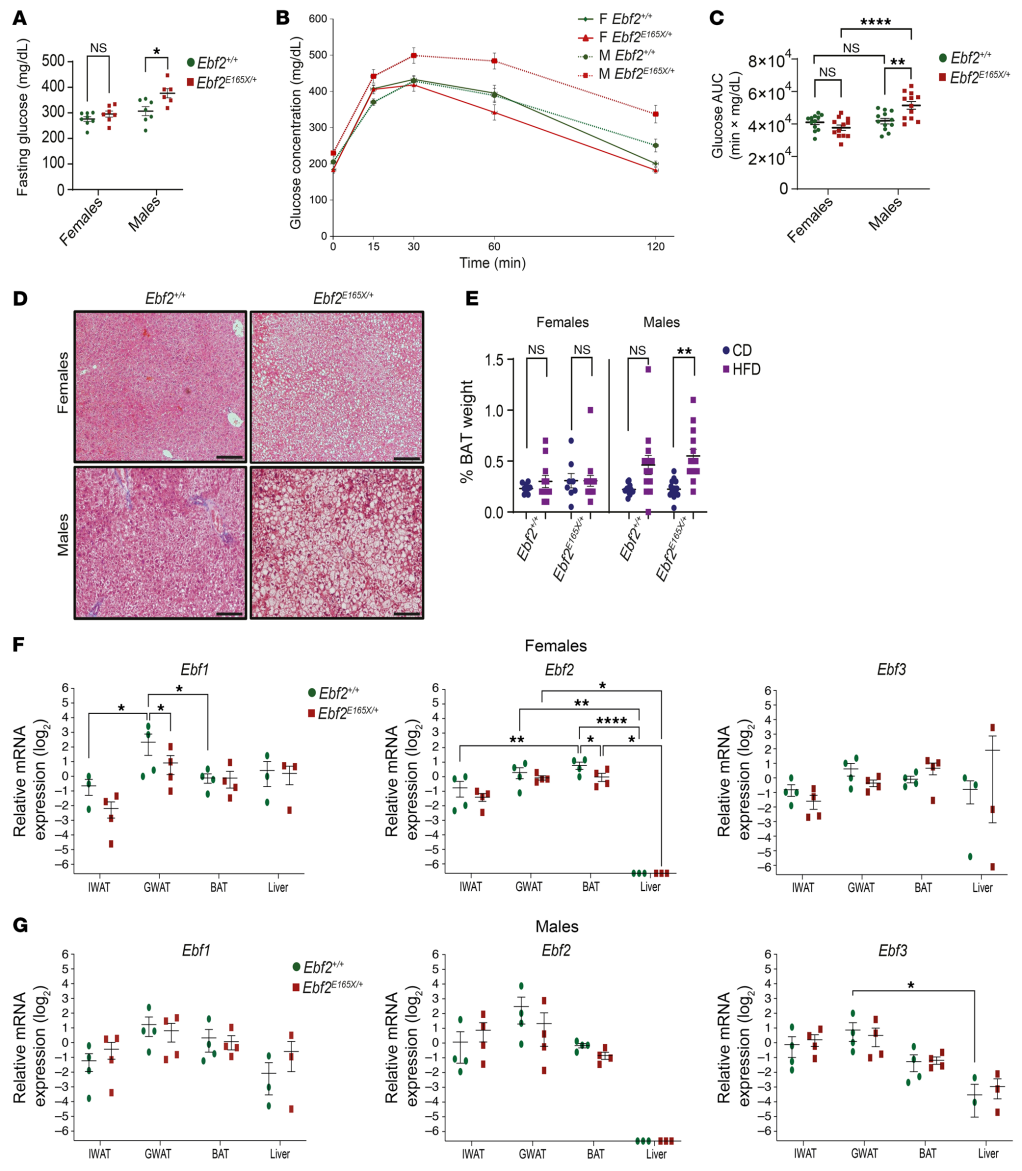
*EBF2* p.E165X worsens HFD-associated metabolic phenotypes. (**A**) Serum glucose after 8 weeks of a HFD (*n* = 6–8). (**B** and **C**) IPGTT and AUC (*n* = 8). (**D**) Masson’s trichrome–stained images of liver. Scale bars: 100 μm. (**E**) Percentage of BAT weight (*n* = 8–15). *P < 0.05, **P < 0.01, and ****P < 0.0001 by 2-way ANOVA with Tukey’s test (**A**, **C**, and **E**–**G**) and 2-tailed unpaired Student’s t test (**B**). Data indicate the mean ± SEM. Each dot represents 1 mouse. See also Supplemental Figures 4–6.

We next examined whether the *Ebf2* p.E165X variant causes metabolic impairment in mice fed a HFD. Fasting serum glucose levels were elevated in male *Ebf2^E165X/+^* mice after 2 months of a HFD compared with *Ebf2^+/+^* mice (Figure 4A). Moreover, the IPGTT demonstrated impaired glucose clearance in *Ebf2^E165X/+^* male mice (Figure 4, B and C). During the IPGTT, insulin levels were not different among the groups (mean with SEM, at 0 minutes, female *Ebf2^+/+^* 2.23 ± 0.41 ng/mL vs. *Ebf2^E165X/+^* 2.27 ± 0.47, *P* = 0.94; male *Ebf2^+/+^* 3.56 ± 0.47 vs. *Ebf2^E165X/+^* 4.08 ± 0.49, *P* = 0.45; at 15 minutes, female *Ebf2^+/+^* 3.62 ± 0.83 vs. *Ebf2^E165X/+^* 2.82 ± 0.54, *P* = 0.43, male *Ebf2^+/+^* 4.67 ± 0.44 vs. *Ebf2^E165X/+^* 5.09 ± 0.85, *P* = 0.66). Introperitoneal insulin tolerance tests further demonstrated a reduced glucose clearance in male *Ebf2^E165X/+^* mice at 15 minutes; however, no significant difference between the groups was observed at 30 minutes in mice of either sex (Supplemental Figure 6A). These results indicate a subtle but significantly reduced insulin-dependent glucose clearance in *Ebf2^E165X/+^* male mice. The liver showed increased steatosis in *Ebf2^E165X/+^* mice relative to *Ebf2^+/+^* mice in both sexes (Figure 4D). We wondered whether the *Ebf2^E165X/+^* metabolic phenotype was associated with lower energy expenditure, since *Ebf2* regulates BAT development and function (16, 34). *Ucp1* expression in BAT was affected by both genotype and diet in female mice (2-way ANOVA, *P* = 0.045 and 0.035, respectively) but showed no effects in male mice (*P* = 0.95 and 0.08). *Cidea* expression was regulated by diet but not genotype in both sexes (females, *P* = 0.04 and 0.35; males, *P* = 0.01 and 0.68) (Supplemental Figure 6B). BAT mass did not differ between *Ebf2^+/+^* and *Ebf2^E165X/+^* mice fed a CD (Figure 4E), but a HFD significantly increased BAT mass in male *Ebf2*^E165X/+^ mice (Figure 4E). *Ebf2^E165X/+^* mice showed increased lipid droplets in BAT (Supplemental Figure 6C). This difference was further exacerbated by the HFD (Supplemental Figure 6C). We observed no differences in VCO_2_, VO_2_, the respiratory exchange ratio, or energy expenditure between the genotypes at 3 different temperatures: room (22°C), thermoneutral (30°C), and cold (10°C) when analyzed with either a Student’s *t* test after normalizing for lean mass (Supplemental Figure 7, A–C) or by regression analysis using lean mass as a covariate (Supplemental Figure 7, D and E). *Ebf2^+/+^* and *Ebf2^E165X/+^* mice exhibited the expected increase in energy expenditure at 10°C, confirming their similar preservation of thermogenic capacity. EBF family members may have unique and compensatory roles in regulating depot-specific adipose functions. Tissue distribution analysis showed that *Ebf1* and *Ebf2* exhibited sex- and depot-dependent expression (*P* = 0.0013 and 0.0096, respectively), whereas *Ebf3* expression was not depot dependent (*P* = 0.62) (Figure 4, F and G). In *Ebf2^E165X/+^* mice, the depot-dependent expression of *Ebf1* and *Ebf2* seen in females was absent, indicating that the *Ebf2* variant negatively affected sex- and depot-dependent *Ebf1* and *Ebf2* expression (Figure 4F). In male mice, *Ebf1* and *Ebf2* were more highly expressed in GWAT, as in females, but the differences were less noticeable. *Ebf2* expression in the liver was very low in both male and female mice (Figure 4, F and G).

### The EBF2 variant impairs AT metabolic function.

**Figure 5:**
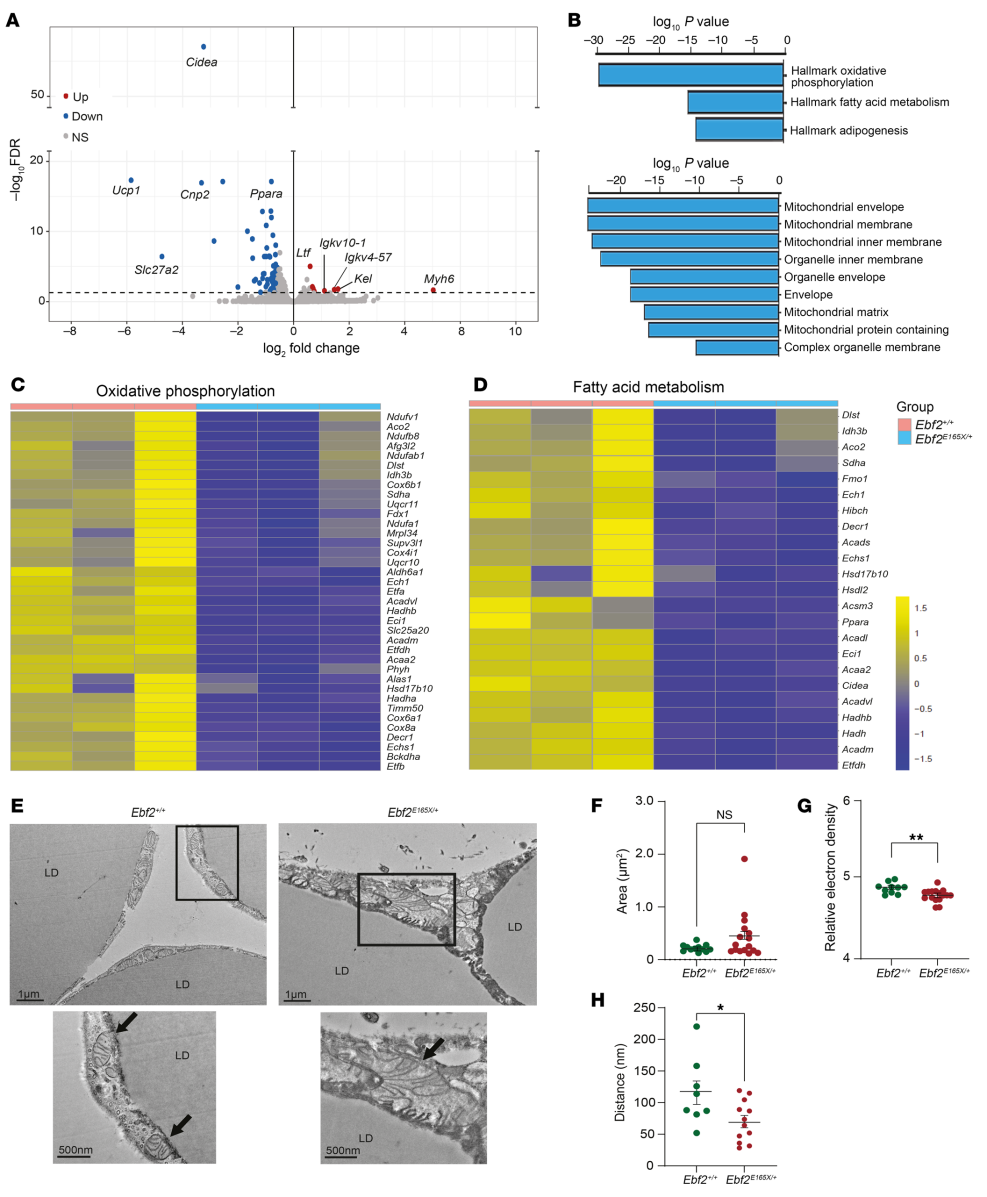
*EBF2* p.E165X alters IWAT metabolic function. (**A**) Volcano plot of differentially expressed genes in 4-week-old male mouse IWAT (*n* = 3/group). (**B**) Downregulated pathways (Hallmark, GO). (**C** and **D**) Heatmaps of genes involved in oxidative phosphorylation and fatty acid metabolism. (**E**) TEM of IWAT with mitochondria (arrows) and lipid droplets (LD). (**F**–**H**) Quantification of mitochondrial area, electron density, and distance to LD. Scale bars: 1 μm and 500 nm (insets) (**E**). *P < 0.05 and **P < 0.01, by 2-tailed, unpaired Student’s t test (**F**–**H**). Data indicate the mean ± SEM. Each dot represents 1 mitochondrion.

To determine the effect of the *Ebf2* nonsense variant on WAT, we used unbiased transcriptome analyses of IWAT isolated from 4-week-old male *Ebf2^+/+^* and *Ebf2^E165X/+^* mice, before the onset of noticeable structural and functional impairments of *Ebf2^E165X/+^* mice (Gene Expression Omnibus [GEO] accession no. GSE288829). We identified 190 transcripts that were differentially expressed in *Ebf2*^E165X/+^ *and Ebf2*^+/+^ IWAT, with an adjusted *P* value of less than 0.05 and a log_2_ fold change of 1.5 or greater. Notably, *Ucp1*, *Cidea*, and *Ppara* expression levels were markedly downregulated in the IWAT of *Ebf2^E165X/+^* mice (Figure 5A). Conversely, the expression of immunoglobulin genes was upregulated in *Ebf2^E165X/+^* mice (Figure 5A). Pathway analysis using iPathwayGuide showed reduced expression of oxidative phosphorylation, fatty acid metabolism, and adipogenesis pathway genes in *Ebf2*^E165X/+^ IWAT (Figure 5B). Consistently, Gene Ontology (GO) cellular component analysis identified reduced expression of mitochondrial genes in the *Ebf2^E165X/+^* IWAT (Figure 5B). Detailed analysis of gene expression in oxidative phosphorylation and fatty acid metabolism pathways showed a reduction of mitochondria-related genes in *Ebf2^E165X/+^* mice (Figure 5, C and D). Complementary gene set enrichment analysis (GSEA) further showed positive enrichment of pathways related to epithelial-mesenchymal transition (EMT), inflammatory response, TGF-β signaling, and PI3K/AKT/mTOR signaling (Supplemental Figure 8A). In contrast, pathways associated with adipogenesis, fatty acid metabolism, oxidative phosphorylation, and cholesterol homeostasis were negatively enriched (Supplemental Figure 8B). Given the decreased mitochondrial gene expression observed in *Ebf2^E165X/+^* mice, we assessed mitochondrial morphology using transmission electron microscopy (TEM). In *Ebf2^E165X/+^* IWAT, we found that mitochondria were more elongated and irregularly shaped with increased variability (*F* test *P* < 0.0001) (Figure 5, E and F). *Ebf2^E165X/+^* IWAT mitochondria frequently exhibited a loss of cristae structures compared with controls, resulting in decreased electron density (Figure 5, E and G). Moreover, *Ebf2^E165X/+^* IWAT mitochondria were positioned closer to lipid droplet membranes (Figure 5, E and H).

### The EBF2 variant impairs white adipocyte differentiation in a cell-autonomous manner.

**Figure 6:**
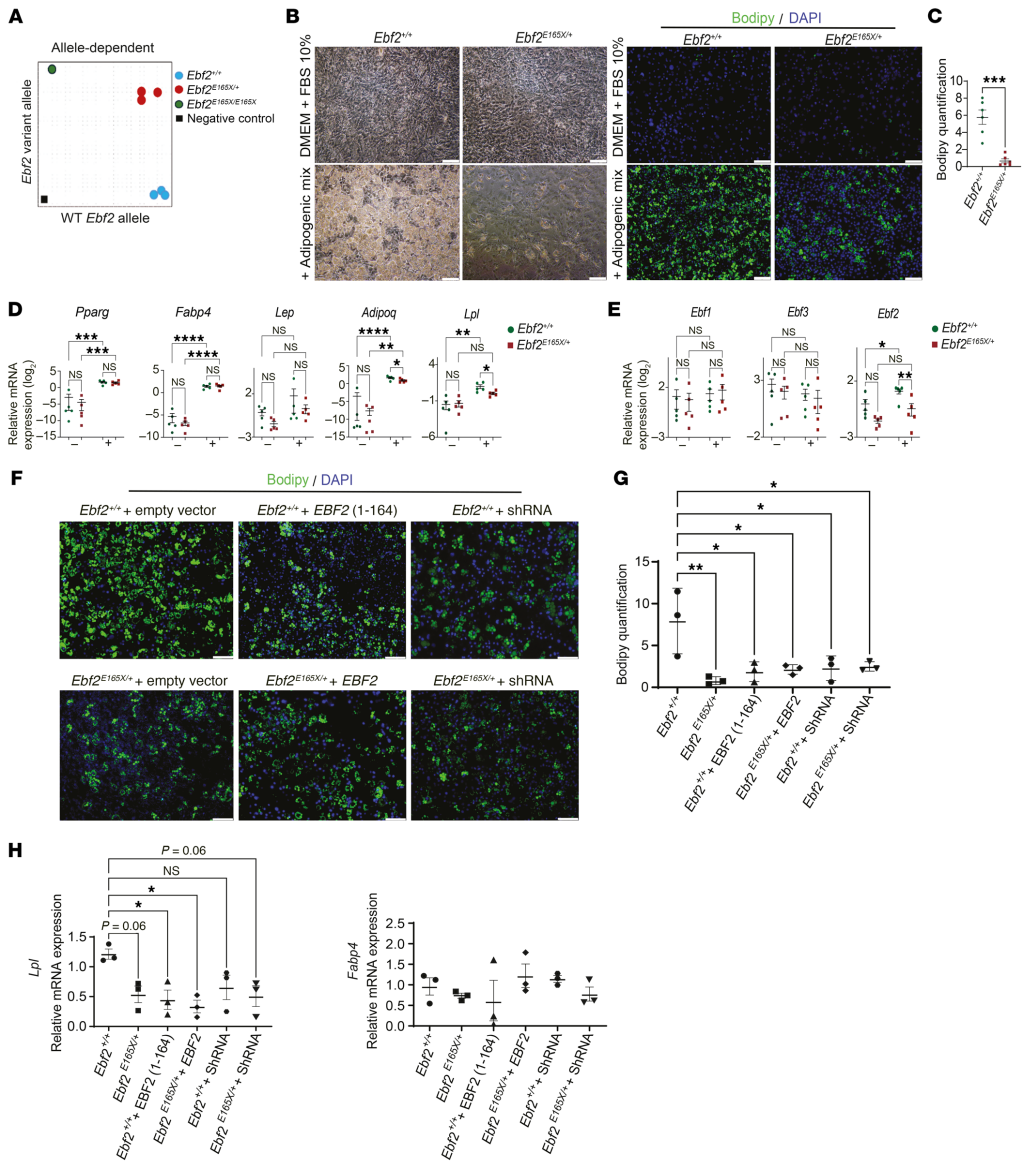
The *EBF2* variant impairs adipocyte differentiation in a cell-autonomous, dominant-negative manner. (**A**) Allele-specific RT-qPCR of IWAT (*n* = 3–5). (**B**) Differentiated IWAT SVF from *Ebf2^+/+^* and *Ebf2^E165X/+^*. BODIPY (green), DAPI (blue). **(C**) Quantification of lipid accumulation (*n* = 6). (**D** and **E**) Expression of adipogenic genes and *Ebf* family members in the SVF before and after differentiation (*n* = 5). (**F**) Effects of truncated and full-length EBF2 and shRNAs in the SVF. (**G** and **H**) Quantification of lipid accumulation and gene expression (*n* = 3). Scale bars: 100 μm (**B** and **F**). *P < 0.05, **P < 0.01, ***P < 0.001, and ****P < 0.0001, by 1 or 2-way ANOVA with Tukey’s test (**C**–**E**, **G**, and **H**). Data indicate the mean ± SEM. See also Supplemental Figures 1 and 7.

In the IWAT of *Ebf2^E165X/+^* mice, WT and variant *Ebf2* transcripts were equally detected with respective TaqMan probes (Figure 6A). We then asked whether cell-autonomous adipocyte defects caused the IWAT phenotype of *Ebf2^E165X/+^* mice. To address this question, we isolated the stromal vascular fraction (SVF) from IWAT depots and induced adipogenesis in vitro. The *Ebf2^+/+^* SVF underwent efficient morphological differentiation into lipid-containing adipocytes (Figure 6B). On the contrary, the *Ebf2^E165X/+^* SVF showed significantly impaired lipid accumulation (Figure 6, B and C). Unlike the IWAT SVF, the BAT SVF of *Ebf2^E165X/+^* mice exhibited an adipogenic capacity comparable to that seen in controls (Supplemental Figure 9, A and B), and BAT-specific genes such as *Cidea* and *Ucp1* were expressed equally between the groups (Supplemental Figure 9C).

*Pparg* and *Fabp4* were expressed at comparable levels in *Ebf2^+/+^* and *Ebf2^E165X/+^* IWAT–derived adipocytes in vitro (Figure 6D). *Lep* expression was too low to be reliably assessed in the in vitro model. However, expression of *Adipoq* and *Lpl* was significantly lower in *Ebf2^E165X/+^* adipocytes (Figure 6D). Given the involvement of other *EBF*s in adipogenesis (8), we examined whether the *Ebf2* variant affected the expression of other EBF members. *Ebf1* and *Ebf3* were expressed at similar levels in *Ebf2^+/+^* and *Ebf2^E165X/+^* IWAT–derived cells (Figure 6E). In contrast, *Ebf2* expression was induced by adipogenesis but was lower in *Ebf2^E165X/+^* cells (Figure 6E). When we induced the expression of *EBF2* (1-164) in the *Ebf2^+/+^* IWAT-derived SVF to assess the potentially dominant-negative effect of the *EBF2* variant on adipogenesis, we observed a significant reduction in adipogenic potential (Figure 6, F and G). The adipogenesis of *Ebf2^+/+^* cells after *EBF2* (1-164) transduction was reduced to a degree similar to that seen in the *Ebf2^E165X/+^* mouse–derived SVF (Figure 6, F and G). Furthermore, full-length EBF2 cDNA could not restore the adipogenic potential of *Ebf2^E165X/+^* cells (Figure 6, F and G), indicating a dominant-negative mechanism rather than a LoF, through which the EBF2 variant inhibits adipogenesis. Silencing of *Ebf2* with shRNA nullified the differences in adipocyte maturation and lipid accumulation between *Ebf2^+/+^* and *Ebf2^E165X/+^* SVFs (Figure 6, F and G), supporting the causal role of the *Ebf2* gene variant in these differences. Expression of the variant *EBF2* (1-164) in *Ebf2^+/+^* cells decreased *Lpl* expression (Figure 6H), and the reduced expression could not be rescued by full-length *EBF2* in *Ebf2^E165X/+^* cells (Figure 6H). However, *Fabp4* expression was unaffected in either *Ebf2^+/+^* or *Ebf2^E165X/+^* cells (Figure 6H). These results suggest that the *EBF2* variant product EBF2 (1-164) disrupts the expression of selective adipocyte genes.

ZNF423 and ZNF521 are multi–zinc finger transcription factors (TFs) that regulate adipocyte differentiation (40, 41). ZNF423 maintains white adipocyte identity in part by antagonizing the brown fat–promoting activity of EBF2 (17, 42). We hypothesized that the truncated EBF2 variant EBF2 (1-164) disrupts white adipocyte differentiation by interfering with other EBF family members and their coregulators ZNF423 and ZNF521 (17, 43). To test this, we compared full-length *EBF2* and *EBF2* (1-164) using synthetic reporters containing EBF- or ZNF-response elements (11, 44, 45). *EBF1–3* increased EBF consensus reporter activity compared with control conditions lacking these drivers, and coexpression of WT or truncated *EBF2* significantly modulated reporter activity (interaction *P* < 0.0001, 2-way ANOVA). Both full-length and truncated *EBF2* enhanced *EBF1*-dependent luciferase activity in a dose-dependent fashion, whereas neither construct produced a consistent effect on *EBF2*-driven reporter activity; a modest increase was observed only at the highest dose of the truncated *EBF2* variant. In the *EBF3*-driven condition, low-dose WT EBF2 was associated with reduced reporter activity, although this effect was not consistently observed across experimental conditions (Supplemental Figure 10A). In ZNF423-binding element reporter assays, both full-length and truncated *EBF2* suppressed reporter activity at a higher dose. In contrast, we observed no suppression in ZNF521 consensus reporter assays at this dose (Supplemental Figure 10B). EBF consensus element reporter activity, which ZNF423 and ZNF521 could also promote, showed greater suppression by the truncated *EBF2* than full-length *EBF2* for ZNF423-driven activity. In contrast, both full-length and truncated EBF2 suppressed ZNF521-driven activity (Supplemental Figure 10C). Together, these findings indicate that the *EBF2* variant does not function as a dominant-negative regulator of EBF family–dependent transcription activity in this assay system, but may differentially influence transcriptional outputs involving ZNF423- and ZNF521-associated transcriptional regulatory activities in a context-dependent manner (11, 44, 45).

### Loss of H3K27Ac binding at key regulatory motifs in Ebf2^E165X/+^ adipocyte precursor cells.

Adipose ECM remodeling is central to AT development (22, 46) and contributes to obesity-induced adipose dysfunction (47, 48) and the establishment of obesogenic epigenetic memory (49). A subset of adipocyte precursor cells (APCs), such as Cd45^−^Cd31^−^Pdgfra^+^Cd9^+^ stromal cells, has been implicated in AT fibrosis (50) through augmented PDGFRA/mTOR signaling (51). To determine whether the change in AT cell composition contributes to the altered AT gene expression profile in *Ebf2^E165X/+^* IWAT, we applied deconvolution analysis to the 4-week-old IWAT bulk RNA-seq data using adipose snRNA-seq data (GSE236580) (49) (Supplemental Figure 11A). This analysis revealed no significant differences in cell-type proportions between *Ebf2^+/+^* and *Ebf2^E165X/+^* IWAT (Supplemental Figure 11B). Likewise, the abundance of Cd45^−^Cd31^−^Sca1^+^Cd9^+^ APCs, assessed by flow cytometry, was similar between the genotypes (Supplemental Figure 11, C and D).

Given the observed cell-autonomous defect in adipogenesis and the potential dysregulation of other EBF family members and interacting zinc TF activities, we hypothesized that the presence of the *EBF2* variant widely disrupts transcriptional networks and impairs the proadipogenic potential of adipose stromal cells. To test this, we isolated SVFs from 4-week-old female *Ebf2^+/+^* or *Ebf2^E165X/+^* mice (*n* = 3 per group). We profiled genome-wide TF motif accessibility using cleavage under targets and tagmentation (CUT&RUN) with H3K27Ac as a probe. *Ebf2^+/+^* SVFs demonstrated the enrichment of a series of TF motifs, including CCCWNGGG (EBF1/EBF2), CACGTG (BMAL1, CLOCK, NPAS2), and ACCACA (RUNX1/RUNX2) in comparison with *Ebf2^E165X/+^* SVFs (Supplemental Table 5). We detected differential H3K27Ac binding in the promoter regions of *Atg4b*, *Rarg*, *Pnp*, and *Far1* (Supplemental Table 6). *Atg4b*, a direct target of *Cebpb*, contributes to adipogenesis by regulating autophagy (52). *Rarg* functions as a nuclear receptor central to the transcriptional control of adipogenesis (53). *Pnp*, which encodes purine nucleoside phosphorylase, is a key regulator of purine metabolism and may influence uncoupling protein 1 (UCP1) activity (54). *Far1* encodes fatty acly-CoA reductase, a peroxisome enzyme essential for the synthesis of ether phospholipids, including plasmalogens (55). Collectively, these findings suggest that epigenetic perturbations induced by the *Ebf2* variant contribute to widespread dysregulation across pathways governing fatty acid metabolism, ECM remodeling, and inflammatory cytokine signaling (56).

### Effects of EBF2 and EBF2 (1-164) on human adipocyte differentiation and gene expression.

**Figure 7:**
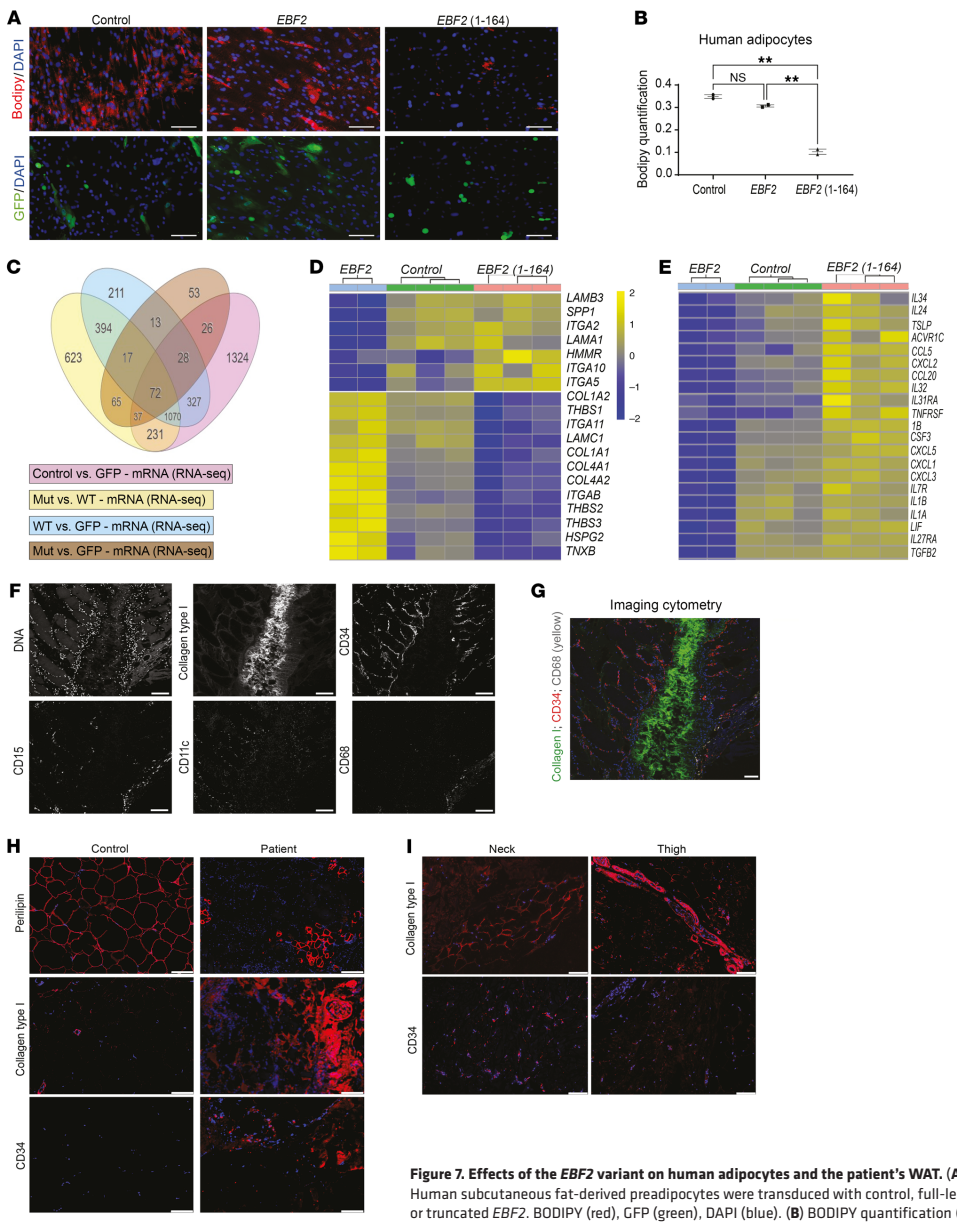
Effects of the *EBF2* variant on human adipocytes and the patient’s WAT. (**A**) Human subcutaneous fat-derived preadipocytes were transduced with control, full-length, or truncated *EBF2*. BODIPY (red), GFP (green), DAPI (blue). (**B**) BODIPY quantification (*n* = 2). (**C**) Venn diagram of RNA-seq of differentially expressed genes. WT, EBF2; Mut, EBF2 (1-164). Mut, mutation. (**D** and **E**) Heatmaps of ECM and cytokine receptor genes. (**F** and **G**) CyTOF images of the patient’s neck WAT showing DNA, collagen I, CD34, CD15, CD11c, and CD68. (**H** and **I**) Immunofluorescence images of patient’s and control WAT depots. Scale bars: 100 μm (**A** and **F**–**I**). **P < 0.01, by 1-way ANOVA with Tukey’s test (**B**). Data indicate the mean ± SEM. Each dot represents a single image.

We reproduced the dominant-negative effect exerted by the truncated *EBF2* nonsense variant in human preadipocytes. Expression of the *EBF2* variant in human preadipocytes decreased adipogenesis, as shown by reduced lipid droplet staining (Figure 7, A and B). We performed unbiased bulk RNA-seq of human preadipocytes transduced with lentivirus constructs of control, full-length EBF2 and truncated EBF2 (1-164), which were induced to differentiate into adipocytes (Figure 7C, GSE288824). *EBF2* and *EBF2* (1-164) led to differential gene expression of PI3K-AKT, ECM, and cytokine–cytokine receptor interaction pathways (Figure 7, D and E), mirroring the changes observed in the IWAT of *Ebf2^E165X/+^* mice. Cells transduced with the *EBF2* (1-164) variant showed reduced expression of *COL1A1*, *COL1A2*, *COL4A1*, *THBS1*, and *TNXB*, along with increased expression of *LAMB3*, *SPP1*, *LAMA1*, *ITGA10*, and *ITGA5* (Figure 7D). Additionally, *EBF2* (1-164) increased the expression of specific cytokines and growth factors, including *IL1A*, *IL1B*, *CXCL2*, *CCL5*, *IL24*, and *TGFB2* (Figure 7E). These findings suggest that the nonsense *EBF2* variant disrupted gene expression in key biological pathways, including the PI3K/AKT/mTOR signaling pathway, ECM remodeling, and cytokine–cytokine receptor interactions, in both mice and humans.

### Fibrotic and inflamed AT in the patient with the EBF2 p.E165X variant.

Given increased numbers of CD34^+^ cells, coupled with excess ECM deposition in *Ebf2^E165X/+^* mice and altered ECM and cytokine expression in human adipocytes, we sought to determine the composition of AT ECM and the immunophenotype in our patient using cytometry by time-of-flight (CyTOF) imaging. CyTOF identified infiltration of neutrophils (CD15^+^ cells), monocytes and macrophages (CD11c^+^ and CD68^+^ cells), and CD34^+^ cells in the patient’s WAT, highlighting the complexity of the stromal cell population in this tissue (Figure 7F). Furthermore, CyTOF imaging revealed significant type I collagen deposition, surrounded by CD34^+^ cells and elevated levels of macrophages (CD68^+^ cells) (Figure 7G). These findings were corroborated by immunofluorescence staining, which showed reduced perilipin 1 positive (PLIN1^+^) adipocytes, increased type I collagen deposition, and an increased prevalence of CD34^+^ cells (Figures 7, H and I). The accumulation of ECM proteins, an increased number of CD34^+^ stromal cells, and the infiltration of myeloid and lymphoid cells are histological features we found to be shared between the *Ebf2* variant mouse model and the patient, underscoring their pathological similarity.

## Discussion

In this study, we identified an *EBF2* p.E165X variant in a patient with atypical PLD, thereby uncovering the critical role of EBF2 in regulating adipogenesis and ECM remodeling. To our knowledge, this is the first report linking *EBF2* dysfunction to PLD syndromes. Advances in genetic technology have identified an expanding number of candidate gene variants that may underlie PLD. However, functional characterization and validation of newly identified gene variants remain challenging, highlighting a translational research gap between clinical and genetic studies of LD syndromes and the broader field of AT biology, as is the case with other rare diseases. Our study provides critical evidence linking the *EBF2* p.E165X variant to impaired adipogenesis and AT function, thereby bridging a crucial gap between the genotype and phenotype in understanding the pathogenesis of LD in this patient.

The *Ebf* family, including *Ebf1* and *Ebf2*, regulates mouse adipogenesis in vitro (8, 57, 58) and plays a key role in adipocyte differentiation and the development of WAT and BAT (15, 16, 59, 60). However, the role of the *EBF* family in human AT development remains undefined. To gather more convincing evidence and address the limitations of the in vitro assays, we developed *Ebf2* p.E165X heterozygous-KI (*Ebf2^E165X/+^*) mice. These mice were fertile and exhibited no discernible defects at birth. However, we obtained very few homozygous KI (*Ebf2^E165X/E165X^*) mice on a mixed genetic background and none on a C57BL/6 background, suggesting that either a complete loss of *Ebf2* or an increased gene dosage of the *Ebf2* p.E165X variant may have caused a lethal phenotype. The *Ebf2^E165X/E165X^* mouse showed restricted WAT development with excess ECM deposition. The lethality of the *Ebf2^E165X/E165X^* mice contrasts sharply with the phenotype of the whole-body *Ebf2*-null mice, which are not lethal but display a wide array of biological impairments in the migration of gonadotropin-releasing hormone (GnRH) neurons (61), peripheral nerve myelination (62, 63), bone development (13), BAT development (16), and WAT beiging (64). These biological phenotypes may explain some of the clinical features of our patient (delayed puberty, scoliosis, hand contracture, pain hypersensitivity). However, unlike global *Ebf2*-KO mice, the *Ebf2^E165X/E165X^* mice showed near-complete neonatal lethality, suggesting that the *EBF2* (1-164) variant had a more severe effect on peri- and postnatal development than would be expected from its LoF. Our study demonstrates that the *Ebf2* variant broadly disrupts H3K27Ac recruitment to multiple TF motifs in adipose stromal cells, highlighting the detrimental effect of the truncated protein on transcriptional networks, which may have contributed to the near-lethal phenotype of *Ebf2^E165X/E165X^* mice.

AT fibrosis or excess ECM deposition is observed in obesity and LD (65, 66). Tissue fibrosis is often viewed as a consequence of inflammation; however, the precise sequence and interplay between inflammation and fibrosis in the development of diabetes remain poorly understood (67). At 4 weeks of age, the AT structure in *Ebf2^E165X/+^* mice appeared identical to that of *Ebf2^+/+^* mice, before the onset of adult WAT expansion. Before post-weaning WAT expansion, the AT was densely packed with numerous small adipocytes, each encased in collagen fibers (22, 46). Between 4 and 16 weeks of age, *Ebf2^+/+^* IWAT displayed uniform hypertrophy of adipocytes, with a diminution of collagens and fibronectin content and a decreasing number of CD34^+^ cells. In contrast, *Ebf2^E165X/+^* mice showed dysmorphic adipocyte hypertrophy with persistent collagen and fibronectin deposition, indicating restricted adipogenesis and impaired ECM remodeling that resulted in IWAT fibrosis (22, 48). These findings suggest that excess ECM deposition in ATs of *Ebf2^E165X/+^* mice occurs during the post-weaning stages of WAT expansion, when adipogenesis and ECM remodeling need to be coordinated.

Mitochondrial biogenesis and function are crucial for the healthy development and expansion of AT (68). As mentioned earlier, at 4 weeks of age, *Ebf2^E165X/+^* IWAT did not exhibit noticeable differences from *Ebf2^+/+^* IWAT, as observed through H&E and immunofluorescence staining of CD34^+^ cells and ECM proteins. However, bulk RNA-seq of IWAT revealed mitochondrial gene suppression and reduced expression of *Ucp1*, *Cidea*, and *Ppara*. These genes are expressed in mitochondria-rich brown and beige adipocytes, suggesting that the *Ebf2* nonsense variant may interfere with mitochondrial function (16, 42, 64). This indicates that early perinatal AT development may not require extensive mitochondrial involvement. During the pre-weaning phase, mice primarily rely on free fatty acids rather than carbohydrates as an energy source for growth, and adipocytes can directly absorb free fatty acids (69). Following weaning, WAT expands with de novo lipogenesis as mice are transitioned to a carbohydrate-rich diet, with the induction of enzymes involved in fatty acid synthesis, such as acetyl CoA carboxylase (*Acc*) and ATP citrate lyase (*Acly*) (70). Notably, 4-week-old *Ebf2^E165X/+^* IWAT had comparable expression levels of *Fatp1* (*Slc27A1*), *Cd36*, *Ascl1*, *Fabp4*, and *Lipe*, which are involved in free fatty acid uptake and retention. Moreover, the expression of *Insr* and *Igf1r*, key regulators of AT expansion (71), was comparable between 4-week-old *Ebf2^E165X/+^* and *Ebf2*^+/+^ mice. However, maladaptation to a carbohydrate-rich diet might have occurred in *Ebf2^E165X/+^* mice because of the suppressed expression of mitochondrial genes, including *Aco2* (72), *Hadha*, and *Hadhb* (73). Decreased oxidative phosphorylation, driven by downregulated mitochondrial genes, may have led to ineffective de novo lipogenesis and impaired adipocyte maturation (74). Despite the suppressed mitochondrial gene expression, bulk RNA-seq data showed abundant expression of mitochondrially encoded genes, including *Mt-Atp8*, *Mt-Co1*, *Mt-Cytb*, and *Mt-Nd1* in *Ebf2^E165X/+^* IWAT. Consistently, TEM examination of IWAT did not reveal a significant decrease in mitochondrial numbers. However, the IWAT mitochondria displayed morphological abnormalities and association with lipid droplets. These findings suggest that the AT mitochondrial dysfunction in postnatal IWAT in *Ebf2^E165X/+^* mice was likely caused by the selective downregulation of nucleus-encoded mitochondrial genes due to EBF2 dysfunction.

AT plasticity, homeostasis, and metabolic consequences can be assessed in vivo through HFD challenges (67). *Ebf2^E165X/+^* mice on a 45% HFD demonstrated hypertrophic adipocytes of variable sizes, excess ECM deposition, tissue macrophage infiltration, liver steatosis, and glucose intolerance compared with *Ebf2^+/+^* mice. These AT and metabolic phenotypes resembled clinical presentations of patients with PLD (2, 35, 75). Moreover, serum leptin and adiponectin levels became markedly lower in *Ebf2^E165X/+^* mice than in *Ebf2^+/+^* mice after the HFD challenge, suggesting that the diet was a critical modifier of genetically predisposed ATs. These findings align with the diagnostic challenges of PLD, as these patients often present to clinics only after developing prediabetes, metabolic dysfunction–associated steatohepatitis (MASH), dyslipidemia, and kidney disease as adults. Notably, when these mice were maintained on a CD, they did not show metabolic deterioration at 16 weeks of age despite the disrupted AT structure. This suggests that dietary modification potentially mitigates adipose dysfunction and metabolic decline in PLD caused by EBF2 dysfunction.

Mouse models may not fully capture the heterogeneity of human AT depots and their functions, particularly subcutaneous AT (13, 61, 64). However, *Ebf2^E165X/+^* mice showed restricted AT expansion characterized by dysmorphic adipocyte hypertrophy and excess ECM deposition, particularly in the IWAT of males and females, increased macrophage infiltration, and glucose intolerance in male mice. Our study included a sufficient number of female mice and confirmed restricted adipogenesis, excess ECM deposition, and decreased leptin and adiponectin levels in *Ebf2^E165X/+^* female mice. However, C57BL/6J female mice are resistant to HFD-induced obesity and diabetes, most likely due to increased energy expenditure compared with male mice and the protective effect of estrogen (76). Prolonged HFD feeding and ovariectomy or estrogen receptor blockade may further improve the utility of *Ebf2^E165X/+^* female mice as disease models for women with PLD syndromes (2). Detailed examination of sexual dimorphism, the effects of aging, and various dietary interventions may further refine the utility of *Ebf2^E165X/+^* mice as a PLD animal model (77).

Our findings align with the biological role of *EBF2* in humans, as suggested by several lines of existing evidence. *EBF2* marks a subset of human adipocytes that correlates with increased BMI (18). An *EBF2* common variant was associated with visceral AT mass assessed with MRI (20). Our investigation of published and publicly available databases suggests close associations between *EBF2* common variants and metabolic traits (diabetes, hypertension, and hypertriglyceridemia), overlapping with those of metabolic syndrome (31–33, 75). LD-like abnormal fat distribution and gene polymorphisms associated with LD syndromes were observed in subclusters of individuals with diabetes (34) and cardiovascular diseases (35, 78). Consistent with these SNP association studies, the LD-associated metabolic deterioration observed in our patient with the pathogenic *EBF2* variant supports a critical role for EBF2 in maintaining metabolic homeostasis. Another *EBF* family member, *EBF1*, is expressed at higher levels in functional, hyperplastic adipocytes relative to hypertrophic adipocytes in humans, and the EBF-like binding motif is enriched in adipogenic genes, such as *PPARG*, *NCOR2*, *LIPE*, *PNPLA2*, *PLIN1*, and *CIDEC* (15).

Our in vitro studies indicate that the *Ebf2* p.E165X variant impaired adipocyte lipid accumulation in a cell-autonomous and dominant-negative manner despite the presence of other *Ebf* family members, including *Ebf1* and *Ebf3*. In contrast, reporter assays using a synthetic EBF consensus element in a heterologous cell line did not reveal a dominant-negative effect of the truncated EBF2 protein on EBF1- or EBF3-dependent transcriptional activation. These findings suggest that the pathogenic effects of the *EBF2* variant are unlikely to reflect direct inhibition of EBF family DNA-binding activity, but instead arise from context-dependent disruption of the broader transcriptional regulatory programs that govern adipocyte differentiation, including ZNF423 and ZNF521. The broader effect of the *EBF2* variant was indeed supported by the significantly altered chromatin accessibility, as assessed by H3K27Ac enhancer binding.

Dominant-negative variants of TFs contribute to the pathogenesis of a broad spectrum of diseases (79), including familial PLD, type 3 (FPLD3) caused by PPARG variants (80), combined pituitary hormone deficiency (CPHD) associated with POU1F1 (PIT1) variants (81), and generalized thyroid hormone resistance (GRTH) due to thyroid hormone receptor beta (THRB) variants (82). Emerging therapeutic strategies include the targeted degradation of pathogenic variant proteins using proteolysis-targeting chimeras (PROTACs) (83), suppression or splicing modulation of variant mRNAs with antisense oligonucleotides (ASOs) (84), and ex vivo gene editing followed by transplantation of patient-derived adipose stem cells (85). These therapeutic modalities need to be explored and tested for the prevention and treatment of LD syndromes caused by harmful variant proteins.

Although our study showed a genetic causality of LD by in vivo and in vitro modeling, it is limited by its reliance on a single family. However, the strength of existing genetic evidence from large databases and the previous linkage of the gene to adipogenesis are all supportive and reproducible findings. Moreover, our mouse model is a whole-body KI one; therefore, the *EBF2* p.E165X variant may have exerted additional biological effects in cell types other than adipocytes. Nonetheless, our in vitro study with primary SVFs showed that the *EBF2* p.E165X variant had a deleterious effect on adipocyte differentiation in a cell-autonomous manner, with variant transcript levels comparable to those of WT *Ebf2* in our KI mouse model. However, because of the unavailability of the antibody that detects the N-terminal portion of EBF2, we were not able to demonstrate the presence of the truncated EBF2 variant protein in this study. While it is interesting that our patient has additional phenotypic features that may be linked to EBF2 function, such as hypogonadotropic hypogonadism, bone and skeletal developmental abnormalities (including hand contracture and scoliosis), umbilical hernia, and Alport-like kidney pathology, we did not examine these organ systems in detail in the KI mouse model.

In conclusion, our in vitro and in vivo investigations indicate that EBF2 dysfunction caused by the *EBF2* p.E165X variant compromises AT expansion and remodeling, ultimately leading to metabolic dysregulation. The discovery of a pathogenic *EBF2* variant in our patient helped us unravel the indispensable role of EBF2 in healthy AT expansion and function.

## Methods

### Sex as a biological variable.

Our study examined male and female animals, and similar findings are reported for both sexes. We considered sex a modifier wherever possible. When our study examined the interaction between genotypes and diet, the effect was analyzed in female mice and male mice, respectively.

### Human participants.

The proband has been followed at the University of Michigan since 11 years of age and participated in the LDLync natural history study (NCT03087253) and prior phenotyping/biopsy studies (NCT01679197, NCT00596934). Clinical data were extracted from medical records and the LDLync registry. DNA analyses were conducted through our tissue and blood biorepository (IRBMED: HUM00062732). Healthy AT was obtained from a 33-year-old female donor (IRBMED: HUM00174659).

### Mouse model.

*Ebf2^E165/X^*-KI mice were generated on a C57BL/6J background (strain no. 000664, The Jackson Laboratory) using CRISPR/Cas9 technology at the University of Michigan Transgenic Core.

### Cell cultures.

3T3-L1, HEK293, and human subcutaneous preadipocytes were cultured in DMEM with 10% FBS. Adipogenesis was induced in 3T3-L1 cells with dexamethasone, insulin, and IBMX, or in human preadipocytes and SVFs with insulin, rosiglitazone, and T3.

### Human genetic sequencing.

Exome sequencing (Roche NimbleGen, version 3.0, HiSeq2000, ~50×) and genome sequencing (PCR-free HiSeq X Ten, ~30×) were performed at the University of Michigan and the Broad Institute, respectively. Reads were aligned to hg38 with BWA, processed with Picard/GATK, and variants were called with HaplotypeCaller, filtered with variant quality score recalibration (VQSR), and annotated with variant effect predictor (VEP). Structural variants were detected with GATK-SV, mitochondrial DNA (mtDNA) variants with gnomAD-mitochondria and MitoSAlt, and tandem repeat expansions with ExpansionHunter, version 5.

### Molecular assays.

Standard protocols were used for SVF isolation, RNAi and lentiviral transduction, luciferase reporter assays, reverse transcription quantitative PCR (RT-qPCR), immunofluorescence, lipid droplet staining, histology, TEM, RNA-seq, and CUT&RUN (anti-H3K27Ac; analysis with Bowtie2, MACS2, and HOMER). Details, oligonucleotide sequences, and antibody information are provided in Supplemental Methods and Supplemental Tables 7–9.

### Statistics.

Experiments included 3 or more biological replicates. Data in the figures are shown as the mean ± SEM. Comparisons were performed using a 2-tailed Student’s *t* test or 2-way ANOVA with Tukey’s post hoc test (GraphPad Prism 10). Sex was included as a modifier in the animal studies. A *P* value of less than 0.05 was considered significant.

### Study approval.

All human protocols were IRB approved and conducted with written informed consent. The University of Michigan IACUC approved the animal studies. Written informed consent was obtained from the patient for the use of the photographs shown in Figure 1A.

### Data availability.

RNA-seq data are available in the GEO database (GSE288829, GSE288824). All other data are provided in the main text or supplemental materials. All values presented in the figures are provided in the Supporting Data Values file.

## Authors contributions

MCFF and DG are co–first authors. MCFF conducted experiments, analyzed and collected patient data, organized figures for the initial submission, and drafted the manuscript. DG performed experiments, collected and analyzed animal data, organized figures for the revision, and co-wrote the manuscript. The first authorship order was determined by mutual agreement of the authors. LP analyzed genome sequencing data and reviewed drafts. RKV and EDB performed animal experiments and data collection. MOL, HLR, CG, KR, and MSU provided variant interpretation and manuscript review. AMDR contributed to experiments and to discussions of results. AN collected patient data. PS provided experimental guidance, participated in results discussions, and edited the manuscript. EAO conceived the project, assembled the team, coordinated collaborations, supervised patient studies, provided clinical care, secured funding, reviewed analyses, and co-wrote the manuscript. THC designed and supervised laboratory experiments, curated data, performed final analyses, and co-wrote the manuscript. All authors critically reviewed and approved the manuscript. EAO and THC share responsibility for data integrity.

## Funding support

This work is the result of NIH funding, in whole or in part, and is subject to the NIH Public Access Policy. Under this federal funding, the NIH has been authorized to make the work publicly available in PubMed Central.

- Clinical phenotyping was supported by NIH grant R01-DK088114 (EAO) and the Lipodystrophy Research Fund at the University of Michigan (Sopha Family, Baker Family, Rosenblum Family, White Point Foundation of Turkey).
- RNA-seq was partially supported by NIH grant R01-DK125513 (EAO).
- Infrastructure support was provided by NIH grants 2P30DK089503, 5P30DK020572, UL1TR002240, and the Caswell Diabetes Institute.
- Whole-genome sequencing was provided by the Broad CMG, supported by NIH grant K23DK114551, National Human Genome Research Institute (NHGRI) grants UM1HG008900 and R01HG009141 (with additional support from the National Eye Institute), NIH and the National Heart, Lung, and Blood Institute [NHLBI], NIH), and a Chan Zuckerberg Initiative grant (DAF2019-199278).

## Supplementary Material

Supplemental data

Supplemental table 1

Supplemental table 2

Supplemental table 5

Supplemental table 6

Supporting data values

## Version 1 01/30/2026

In-Press Preview

## Version 2 03/16/2026

Electronic publication
