# Supplementary material for: EBF2 variant identified in a patient with atypical partial lipodystrophy causes adipose fibrosis and dysfunction
Source: J Clin Invest. 2026 Jan 29;136(6):e192737. doi: 10.1172/JCI192737 (PMC12987656; doi:10.1172/JCI192737)
Supplement: Supplemental data [file jci-136-192737-s284.pdf]

Supplemental information

Supplemental Methods

Human genetic sequencing

Whole exome sequencing (WES) was undertaken at the University of Michigan Sequencing Core. DNA was fragmented to 250bp using standard Covaris sonication. Fragmented DNA was then prepared as a standard Illumina gDNA library using IntegenX reagents on the Apollo instrument, where the fragments were end-repaired, A-tailed, and adapter-ligated. Then, the samples were PCR amplified. Libraries were checked for quality and quantity and then pooled to capture four samples per pool. Each pool was then subjected to exome capture (enrichment) using the Roche NimbleGen SeqCap EZ v3.0 according to the manufacturer's protocols (Roche NimbleGen, Indianapolis, IN). The SeqCap EZ Human Exome Library v3.0 covers 64 Mb of coding exons and miRNAs. Samples were run on 4 lanes achieving 50X mean coverage on the HiSeq2000 sequencer (Illumina, San Diego, CA). Whole genome sequencing and data processing were performed on the Genomics Platform at the Broad Institute of MIT and Harvard. PCR-free preparation of sample DNA (350 ng input at >2 ng/ul) is accomplished using Illumina HiSeq X Ten v2 chemistry. Libraries are sequenced to a mean target coverage of >30x. Genome sequencing data were processed using a Picard-based pipeline that included base quality score recalibration and local realignment at known indels. The BWA aligner mapped reads to the human genome build 38. Single Nucleotide Variants (SNVs) and insertions/deletions (indels) are jointly called across all samples using the Genome Analysis Toolkit (GATK) HaplotypeCaller package version 4.0. Default filters were applied to SNV and indel calls using the GATK Variant Quality Score Recalibration (VQSR) approach. Annotation was performed using a Variant Effect Predictor (VEP). GATK-SV (1) was used to detect structural variants (SVs) annotated with the GATK SVAnnotate tool. Mitochondrial DNA (mtDNA) single nucleotide and minor indel variants were called from GS data using the gnomAD-mitochondria pipeline (2), and large mtDNA deletions were called by MitoSAlt (3). ExpansionHunter v5 was used to screen for known disease-associated tandem repeat expansions (TREs) (4).

Human genetic analysis

The variant call set was uploaded to *seqr* (5) for collaborative analysis. The Broad CMG analysis team developed four standard variant filtration searches (5) based on the suspected mode of inheritance, reported pathogenicity in ClinVar, type of variant, frequency in population databases, and variant call quality. We utilized these standardized searches to generate a list of variants for review. Next, we assessed candidate genes for genotypic and phenotypic matches to known disease genes using Online Mendelian Inheritance in Man (OMIM) and published manuscripts. As no strong candidates were identified in known disease genes, we then screened high-impact variants in genes of uncertain significance (GUS). Among them, we identified a stop-gained variant in the *EBF2* gene. Published animal and cell culture studies implicated the *EBF2* transcription factor in brown fat adipogenesis, hypogonadotropic hypogonadism, and peripheral neuropathy (6, 7). The *EBF2* gene was then submitted to the Matchmaker Exchange, but no other individuals with pLoF variants and similar phenotypes were identified.

Summary of EBF2 not_lof/likely_not_lof variants (Supplemental Table 2)

Out of 57 pLoF variants, 25 (44%) were not predicted to result in NMD upon manual curation.
In-frame splicing effects for canonical splice variants (28% of all not_LoF): includes both creation of a cryptic essential splice rescue and in-frame exon variants for predicted LoF (loss of function) variants that are expected to impact splicing. In-frame cryptic splice sites result in an in-frame indel that does not result in NMD. In-frame cryptic splice sites that resulted in partial intron retention were assessed for the inclusion of termination codons in the intronic sequence retained, which could result in truncation of the protein. If there was a prediction of in-frame exon skipping and the exon was>25% of the gene's coding sequence, the variant was considered to result in loss of function. Splicing predictions were assessed by both spliceAI and pangolin using the spliceAI lookup tool (https://spliceailookup.broadinstitute.org/).
Genotyping errors (4% of all not_lof): genotyping flags are tagged when variants have a skewed allele balance, low read depth, or low genotype quality. Variants are given a verdict of likely_not_lof for genotyping errors when genotype quality is <30; OR the read depth is <15, AND allele balance is <25% or >75%.
Not biologically relevant (36% of all not_lof): pLof variants are considered to have uncertain biological relevance when NMD is predicted in at least one transcript, but their overall effect on the protein is not expected to have a biological impact. Variants were flagged as not biologically relevant when they were present in only a minority of GENCODE transcripts, showed weak exon conservation, and had a low proportion

expressed across transcripts (pext). Variants are considered low pext when the mean pext value of the exon that contains the variant is =20% of the maximum pext across the gene. All of these variants were also not annotated as pLoF in the MANE transcript for the gene (ENST00000520164.6).

Last exon (32% of all not_lof): Nonsense or frameshift variants that terminate in the last exon of a gene typically escape NMD and result in a truncated protein. pLOF variants are assigned a not_lof verdict when they terminate in the last exon or the last 50 bp of the penultimate coding exon.

Homopolymers (8% of all not_lof): Homopolymer repeat regions are enriched for false positive indels that may actually be the result of technical errors during PCR amplification that result in an inaccurate repeat length. Therefore, frameshift variants that fall within homopolymer repeats are given a likely-not LoF verdict.

Knock-in mouse model

The CRISPOR algorithm (8) was used to identify two guide RNA targets (sgRNA) predicted to remove the chromosome in exon 7 of *Ebf2*. sgRNA C359A targets the 5' region of the exon with 5' TTGGGGTCCCGAGGCTACCT PAM (AGG) 3', and C359Y targets the 3' region of the exon with 5' CAGCCAGGATTTCCTCATGT PAM (TGG) 3'. Phosphorothioate-modified sgRNAs were synthesized by Integrated DNA Technologies (9, 10). The sgRNA (60 ng/ul) was complexed with HiFi Cas9 protein (50 ng/ul) from IDT (11) and individually tested to determine if ribonucleoprotein (RNP) complexes cause chromosome breaks in mouse zygotes. RNPs were microinjected into fertilized mouse eggs. Eggs were cultured until they developed into blastocysts. DNA was extracted from individual blastocysts for analysis. PCR with primers spanning the predicted cut site was used to generate amplicons for Sanger sequencing (12). Amplicons were produced with C359A forward primer: 5' TGGTCTATTTTCTACATTCTCGCACCTTT 3' and C359A reverse primer 5': AGGAAGGAAAGAAACTGAAGAGCAGAAAT 3'; 514 bp amplicon, C359Y forward primer: 5' CCAGCTTCAGATTGAGGGTTTGAAAAATA 3' and C359Y reverse primer 5': CCACAAAACACGGTTTTTAACAATGTCAC 3'; 764 bp amplicon. Sequencing electropherograms of amplicons from individual blastocysts were evaluated to determine if small insertions/deletions caused by non-homologous end joining (NHEJ) repair of chromosome breaks were present (13). sgRNA C359A and C359Y were found to induce chromosome breaks, and all guide high specificity scores (Cutting Frequency Determination Score, 89 and 83 (8), Moreno-Mateos 58 and 43, respectively (14)). The CRISPR reagents were mixed with a synthetic long single-stranded DNA donor (5 ng/ul, IDT) and were microinjected into fertilized mouse eggs produced by mating superovulated C57BL/6:SJL (B6SJLF1) F1 Hybrids female mice (Jackson Laboratory stock no. 100012) with B6SJLF1 male mice as described (15). CRISPR/Cas9 microinjection of zygotes produced potential founder mice. The sequence of the long single-stranded oligonucleotide DNA donor is:
CTGAGTGAGTTTCCCAGCTTCAGATTGAGGGTTTGAAAAATAAATAGAACATTGTTGCAAAGTTTGCTGTG
ATGCAGCTTGGGGTCCCGAGGCTACCTAGtCTGTTTCCAGGTGTTCTTACTGCAGTAGGCTTGTGGAGCC
TTTTTGGTTTAACGCGGTTTCTCTCACACCATCTTGACTCCTTCCCGTTCTACAGTCGATGCTGTtAGAAGA
AGAGCTGCGGGAACCGGAACGAGACCCCTTCAGACCCGGTCATCATCGACAGGTAGGGGAATGTCGCC
GCCATTTCTGCTCTTCAGTTTCTTTCCTTCCTGAGTCTCCACTCTGAGGACTGATCCTGGAGGACTTCTGC
TGTAAGCTTGGCTTTGTATTTTCTACAAGGCACGAAGCTCCTGCAGCCAGGATTTCCTCATGTTGtCGATGT
CTCTGGAAAGAAATCAGGGCCTTCCTTTTGTTATCCTTTATCCACTCCCTTATTTAATCTTTGGTGTTTTGC
GATGTGACTTTGACAGCCCAGGTTG. Primers used for genotyping were forward 5' TGGTCTATTTTCTACATTCTCGCACCTTT 3' and reverse 5' CCACAAAACACGGTTTTTAACAATGTCAC 3'. Amplicons were purified and submitted for Sanger sequencing. Sequencing electropherograms of amplicons from mice were evaluated to identify peaks consistent with the designed modification. TOPO TA cloning was then performed on DNA from animals with evidence of the changes introduced to confirm the modification. G0 founders were then bred to C57BL6/J (Jackson Laboratory stock no. 000664), and genotyping was repeated in the offspring to confirm germline transmission.

Mice intraperitoneal glucose tolerance test (IPGTT) and insulin tolerance test (ITT) procedures and analyses

An intraperitoneal glucose tolerance test (IPGTT) was performed at the University of Michigan Animal Phenotyping Core (Director: Nathan Qi) on week 16 of CD or week 8 of HFD after a 5-hour fast. Glucose (25%) was given via Intraperitoneal injection (IP) at 1.0g/kg. Blood samples were collected before and after the injection at time 0, 15, 30, 60, and 120 minutes via tail vein bleeding. Blood glucose levels were measured with a glucometer (AcuChek, Roche), and plasma insulin levels were determined using a rat/mouse insulin ELISA kit (Millipore, Cat# EZRMI-13K). Animals were restrained repeatedly for less than a minute while blood

samples were collected. The total area under the curve (AUC) for glucose was calculated using the trapezoidal rule (16). Insulin tolerance tests (ITT) were performed with 16-week-old mice after two months of HFD feeding. Mice were fasted for 5–6 h before testing, and baseline blood glucose was measured from tail vein samples. Human insulin (0.75 U/kg body weight, intraperitoneal) was administered, and blood glucose was monitored at 15, 30, 60, and 90 min using a handheld glucometer. Data were analyzed as absolute glucose values, percent change from baseline, and area under the curve (AUC).

Assessment of body composition in mice
Body composition (lean and fat mass) was assessed before sacrifice by an NMR-based analyzer (EchoMRI, 4in1-900).

Measurement of serum parameters in mice
Blood samples from the mice were obtained from the vena cava after a 5-hour fast, before sacrifice. The samples were left standing for 20 minutes and then centrifuged for 20 minutes at room temperature, 3,000 g, to separate the serum for biochemical assay. Insulin and leptin were measured using a Mouse Adipokine Magnetic Bead Panel (MADKMAG-71K, Millipore-Sigma). The adiponectin was measured using Mouse Adiponectin Single Plex Magnetic Bead Panel (MADPNMAG-70K, Millipore-Sigma). The glucose and triglyceride levels were measured by the Chemistry Laboratory (Michigan Diabetes Research Center-MDRC) using a Glucose Hexokinase assay kit (Cat#GL8319, Randox Laboratories) and a Triglycerides (GPO-PAP) assay kit (Cat# TR8332, Randox Laboratories), respectively.

Mouse calorimetry studies
Sixteen-week-old *Ebf2*$^{+/+}$and *Ebf2* $^{p.E165X/+}$ mice were placed in the indirect calorimetry system (Promethion System, Sable) at the Michigan Animal Phenotyping Core to measure energy expenditure, in-cage activity, food and water intake, under room temperature 22°C (~2 days), 30°C (1 day), and 10°C (1 day) conditions over four consecutive days. Oxygen consumption (VO2), carbon dioxide production (VCO2), and spontaneous motor activity were measured using the Promethion Comprehensive, High-Resolution Behavioral Analysis System (Sa Systems International), an integrated open-circuit calorimeter equipped with an optical beam activity-monitoring device. Mice were weighed each time before the measurements and individually placed in designated mouse cages (Model 3721; 8.1 x 14.4 x 5.5 in.) with free access to food and water. The study was carried out in an experimental room set at 20-23 °C, 30 °C, and 15-10 °C, with a 12:12-hour light cycle (6:00 PM-6:00 AM). The measurements were carried out continuously for 240 hours. During this time, animals were provided with food and water via the feeding and drinking devices installed in the cage. The Promethion food, water intake & body weight monitoring system features high-precision sensors capable of measuring real-time for mice. The system was routinely calibrated before the experiment using a standard gas (20.5% 02 and 0.5% CO2 in N2). V02 and VCO2 in each cage were sampled sequentially for 30 seconds in a 5-minute interval, and the motor activity was recorded every second in X and Z dimensions. The airflow rate through the chambers was adjusted to maintain an oxygen differential of approximately 0.3% at rest. Respiratory quotient (RQ), or respiratory exchange ratio (RER), was calculated as VCO2 / VO2. Total energy expenditure, carbohydrate oxidation, and fatty acid oxidation can be calculated, respectively, based on VO2 and protein breakdown (usually estimated from urinary nitrogen excretion).

Adipose stromal vascular fraction (SVF) isolation
Adipose stromal vascular fractions (SVFs) were isolated from mice aged between 8 and 12 weeks, both male and female, through a protocol adapted from a previously published (17). After dissection, inguinal and brown adipose tissues were washed with 1% Phosphate buffered saline (PBS1X, ThermoFisher Scientific, Cat#10010-023) minced finely, digested in collagenase type 2 (Worthington, Cat# LS004176, Lakewood, NJ) and collagenase D (Sigma-Aldrich, Cart#11088866001, Mannheim, Germany) diluted to 0.003 g/mL and 0.001 g/mL respectively in serum-free DMEM (ThermoFisher Scientific, Waltham, MA). After 1 hour of incubation at 37°C in a shaking incubator, samples were triturated 3-4 times through an 18-gauge needle and then incubated at 37°C for an additional 5 minutes. Next, collagenase was inactivated with an excess of DMEM containing 10% FBS; the samples were sequentially passed through 100- and 40-μm cell strainers to remove debris. Erythrocyte lysis was achieved by 30-second exposure to hypotonic stress, after which cells were resuspended in PBS and centrifuged at 1700 g for 8 min at 10 °C to obtain a high-density pellet containing the SVF. The SVF thus obtained was resuspended in 3 ml of DMEM supplemented with 10% FBS and 1% penicillin-streptomycin, seeded in 12-well plates, and kept in a humidified incubator at 37°C with 5% CO2 for further experiments.

RNA interference and lentivirus-mediated infection
Two different siRNAs were purchased from ThermoFisher Scientific. The 3T3-L1 cells (ATCC, CL-173) were transfected with 5 nM siRNA targeting *EBF2* (MSS203753 and MSS203754) or control siRNA oligos using RNAi MAX (Invitrogen). The oligonucleotide sequence for each siRNA is listed in **Supplemental Table 3.** The University of Michigan Vector Core prepared lentivirus constructs expressing full-length and truncated versions of the human EBF2 sequence under the pLL promoter. RSV-CMV-eGFP vector and five short hairpin RNAs (shRNAs) in the pLKO-puro vector (Mission shRNA, Sigma-Aldrich) targeting distinct regions of the human and mouse *EBF2* gene. The oligonucleotide sequence for each shRNA and the *EBF2* target is listed in **Supplemental Table 4**. Transduction with lentivirus was conducted when cells reached 40-60% confluency with Polybrene (Sigma-Aldrich, Cat#TR-1003-G) at 4 mcg/mL and 5% FBS overnight. Transduction efficiency was evaluated 48 hours post-incubation by observing the GFP signal. Cells containing shRNA were subsequently selected using Puromycin (Sigma-Aldrich #CatP7255) at 1 μg/mL.

Luciferase reporter assay
For luciferase reporter assays, HEK293 and COS-7 cells (ATCC CRL-1573 and CRL-1651) were transfected with pGL3-based reporter constructs containing EBF-RE, ZNF423-RE, or ZNF521-RE enhancer elements, together with the internal control plasmid pRL-SV40 (Promega). Transfections were performed using Lipofectamine LTX (Thermo Fisher Scientific, Cat#15338030). After 24 hours, cells were lysed in Passive Lysis Buffer, and Firefly and Renilla luciferase activities were quantified using the Dual-Luciferase Assay System (Promega) on a GloMax microplate reader. The following multimerized consensus response elements were employed as respective enhancer elements:
• EBF-RE: 5′-ATTCCCAAGGGAAT gggt ATTCCCAAGGGAAT gggt ATTCCCAAGGGAAT gggt ATTCCCAAGGGAAT gggt ATTCCCAAGGGAAT gggt ATTCCCAAGGGAAT-3′ (18)
• ZNF423-RE: 5′-CCGCCC gggt CCGCCC gggt CCGCCC gggt CCGCCC gggt CCGCCC gggt CCGCCC-3′ (19)
• ZNF521-RE: 5′-GACCACCCA gggt GACCACCCA gggt GACCACCCA gggt GACCACCCA gggt GACCACCCA-3′ (20)
Expression plasmids for human EBF1, EBF2, EBF3, ZNF423, and ZNF521 were obtained from OriGene (RC208736, RC220630, RC21522, RC218703, RC217463).

RNA isolation and real-time quantitative PCR (RT-qPCR) analysis
Cells were harvested and lysed in RLT buffer (RNeasy Lysis Buffer, component of RNeasy Mini Kit, Qiagen, Cat#74104). Tissue samples were homogenized with a Tissue-Tearor homogenizer (Biospec, model 985370) in RLT buffer. RNA extraction was performed using QIAshredder (Qiagen, Cat#79654) and RNeasy Mini Kit following the manufacturer's instructions, and cDNA was synthesized using iScript cDNA Synthesis Kit 100 Rxn (Bio-Rad, Cat#1708891). RT-qPCR was performed using Applied Biosystems TaqMan or SYBR Green Universal PCR Master Mixes (ThermoFisher Scientific, Cat#4304437 and Cat#4309155, respectively) on StepOnePlus Real-Time PCR system (Applied Biosystems, Foster City, CA). RNA expression was analyzed with the standard curve method and normalized to *Rplp0* expression. The Taqman and SYBR Green primers used for RT-qPCR are listed in **Supplemental Tables 7 and 8**, respectively.

Tissue immunofluorescent staining
Deparaffinized tissue sections were incubated in blocking solution PBST (5% BSA and 0.3% Triton X-100) for 1 hour at room temperature and later with primary antibodies (diluted 1:100 in blocking solution) against perilipin (Cell signaling, #3470), collagen type I (Rockland Immunochemicals, Cat#600-401-103-0.1), collagen type VI (Rockland Immunochemicals, Cat#600-401-108-0.1), CD34 (Cell signaling, Cat#26233S), fibronectin (Santa Cruz Biotechnology, Cat#sc-8422), UCP1 (Santa Cruz Biotechnology, Sc-6528) overnight at 4°C. After washing 3 times with PBS, the sections were incubated with secondary antibodies (diluted 1:500 in blocking solution) F(ab')2-Goat anti-Rabbit IgG (H+L) Cross-Adsorbed Secondary Antibody, Alexa Fluor™ 594 (ThermoFisher Scientific, Cat#A11072) or Donkey anti-Goat IgG (H+L) Cross-Adsorbed Secondary Antibody, Alexa Fluor™ 647 (ThermoFisher Scientific, Cat# A-21447) for 1 hour at room temperature, shielded from light. Following three PBS washes, the tissues were stained with DAPI (Invitrogen, Cat#D3571) diluted 1:10,000 in water for 10 minutes, rewashed three times with PBS, and mounted with Fluoromount-G (SouthernBiotech,

cat#0100-01). The sections were visualized under a fluorescent microscope (Olympus IX71, Olympus, Center Valley, PA) with a ×10 objective lens (NA 0.65) at 25°C.

Cell Immunofluorescent staining

After complete adipocyte differentiation, 3T3-L1 cells, human cells, and SVFs were fixed with 4% paraformaldehyde at room temperature for 10 minutes. After washing 3 times with PBS, they were incubated with a blocking solution (PBS with 1% BSA) for 30 minutes at room temperature. The cells were stained with BODIPY 493/503 (ThermoFisher Scientific, Cat# D3922) diluted 1:1000 in BSA/PBST and DAPI for 1 hour at room temperature. After washing three times with PBS, the cells were analyzed by fluorescent microscopy using an Olympus IX71 (Olympus, Center Valley, PA) equipped with a ×10 objective lens (NA 0.65) at 25°C.

Histological analysis

The adipose tissue samples were fixed in 4% paraformaldehyde, embedded in paraffin, and sliced into 5-μm sections. Standard hematoxylin and eosin (H&E) and Masson's Trichome staining procedures were used. Quantification of adipocyte area and number was done on H&E-stained sections using ImageJ software from the NIH version 1.6. IWAT fibrosis (%) was quantified by measuring the blue color density in Masson's trichrome-stained sections using ImageJ software. Four randomly chosen high-power fields from each sample for each mouse (n = 3–4 animals) were analyzed. The average value of blue color density was used as the fibrosis index. Digital images of immunofluorescent-stained samples were acquired using a fluorescence microscope, with the same exposure settings maintained throughout. Acquired images were imported into ImageJ and adjusted for brightness and contrast to ensure optimal visualization of features while maintaining uniform adjustments across images. ROIs corresponding to specific cell populations or tissue areas were outlined and selected using appropriate tools in ImageJ. Thresholding was applied to convert images to binary format, separating areas of interest from the background, with threshold levels adjusted to optimize separation while minimizing noise. Measurements of the mean fluorescence area covered were recorded for each ROI. We quantified lipid droplet accumulation in cell cultures using ImageJ by determining the Bodipy-positive area. We quantified Bodipy by calculating the ratio of Bodipy-positive area to DAPI-positive area.

Bulk RNA-seq and data analysis

RNA samples were quality-controlled by measuring RNA integrity numbers (TapeStation analysis software v3.2, Agilent Technologies). The University of Michigan Advanced Genomics Core performed QuantSeq 3′ mRNA sequencing (Lexogen). Data were pre-filtered to remove genes with 0 counts in all samples. Differential gene expression analysis was performed using DESeq2 (21), using a negative binomial generalized linear model (thresholds: linear fold change >1.5 or <-1.5, Benjamini-Hochberg FDR (Padj) <0.05). Plots were generated using variations of DESeq2 plotting functions and other packages with R version 4.1.3. Annotation data from ENSEMBL 109 were used, and genes were additionally annotated with Entrez GeneIDs and text descriptions. Functional analysis, including candidate pathways activated or inhibited in comparison(s) and GO-term enrichments (22), was performed using iPathway Guide (Advaita) (iPathwayGuide) (23).

CUT&RUN

CUT&RUN was performed using the Cell Signaling Technology CUT&RUN Assay Kit (CST, #86652) following the manufacturer's protocol. Briefly, 0.5–1 × 105 adipose stromal cells were immobilized on concanavalin A beads and permeabilized. Cells were incubated overnight at 4°C with anti–H3K27Ac (D5E4) rabbit mAb (CST, #8173) or rabbit IgG XP® isotype control (CST, #66362). After washing, pAG-MNase was added and activated with $CaCl_2$,and released DNA fragments were purified using QIAquick Gel Extraction Kit. Libraries were prepared and sequenced on an Illumina platform at Advanced Genomic Core of the University of Michigan. Reads were aligned to the mm10 genome using Bowtie2, and peaks were called with MACS2 using matched IgG controls as background. Differential binding motif analysis and gene annotation were performed with HOMER (Hypergeometric Optimization of Motif EnRichment). Differentially expressed genes were defined using an FDR < 0.05 and $\log_2$ fold change > 1.5 as cutoffs.

Deconvolution

Bulk RNA-seq deconvolution was performed in R using a non-negative least squares (NNLS) regression framework. Reference expression profiles were derived from single-nucleus RNA-seq data (GSE236580) (39) which were processed and clustered with Seurat to define major cell populations. Gene identifiers were harmonized with biomaRt, and NNLS (via the nnls package) was applied to estimate relative cell-type

proportions in bulk RNA sequencing samples. These deconvolution estimates were compared with the percentages of cells directly quantified from the bulk RNA-seq clusters.

Quantification of *Ebf2* allele-specific transcripts

We designed two primers (forward primer sequence: CATCTTGACTCCTTCCCGTTCTAC; reverse primer sequence: CGATGATGACCGGGTCTGAAG) to amplify the sequence encompassing the *Ebf2* p165X variant and detect the single-nucleotide difference between wild-type and variant alleles using respective probes (probe 1 sequence: TCGATGCTGTGAGAAGA; probe 2 sequence: AGTCGATGCTGTTAGAAGA; ThermoFisher Scientific, custom Taqman assay, assay ID: ANPR7KU, mEbf2_c493G_T). We performed RT-qPCR using a genotyping protocol in the StepOnePlus Real-Time PCR system.

Transmission electron microscopy (TEM)

Chow-fed Ebf2$^{+/+}$ and *Ebf2*$^{E165X/+}$ mice aged 4 and 16 weeks were sacrificed, and inguinal white adipose tissue was excised. The tissues were cut into fragments <1 mm³ and fixed in 3% glutaraldehyde/3% formalin in 0.1 M sodium cacodylate buffer (CB) at 4°C overnight. The samples were washed three times for 15 minutes each with 0.1 M CB, then treated with 1.5% potassium ferrocyanide [$K_4Fe(CN)_6$] and 2% osmium tetroxide (OsO_4) in 0.1 M CB for 1 hour. Subsequently, the samples were washed three times for 5 minutes each with 0.1 M CB, followed by three 5-minute washes with 0.1 M acetate buffer (AB). The samples were then treated with 2% uranyl acetate (UA) in 0.1 M AB for 1 hour and washed twice for 5 minutes each with 0.1 M AB After an additional 5-minute wash in deionized water (DIW), the samples were dehydrated in a graded ethanol series (30%, 50%, 70%, 80%, 90%, 95%, 100%, 100% dry) followed by 100% acetone for 15 minutes at 4°C in each solution. For resin infiltration, the samples were incubated in a 2:1 acetone: resin mixture for 1.5 hours, a 1:1 mixture for 3 hours, and a 1:2 mixture for 16 hours. This was followed by infiltration in 100% resin under vacuum for 48 hours. The samples were then placed in embedding molds and polymerized at 70°C for 24 hours. Thin sections were cut using an ultramicrotome and placed on bare 300-mesh copper TEM grids. The samples were imaged using a transmission electron microscope (TEM; JEOL JEM-1400 Plus).

Statistics

For H&E, Masson's Trichrome, and immunofluorescent staining, at least 3 samples per group were used. Experiments with SVFs were independently repeated at least three times. All data are shown as mean ± SEM. GraphPad Prism 10 software was used for statistical analyses. When comparing the two groups' means, a Student's t-test with a 95% confidence interval was used. We used a two-way ANOVA followed by post hoc Tukey's multiple comparisons test to analyze the effects of two variables and their interaction. A p-value < 0.05 was considered significant for all statistical tests. Ns denotes not significant. In the animal study, we treated sex as a modifier and analyzed it using two-way ANOVA whenever possible. In our study, the interaction between genotypes and diet was analyzed separately in females and males. For tissue gene expression analysis, we used an unpaired t-test to compare *Ebf2*$^{+/+}$ and *Ebf2*$^{E165X/+}$ mice, separately in females and males, because the standard cDNAs used were different. No data was excluded from the study. The investigators were not blinded for the animal and in vitro cell-based experiments. However, at least two observers measured the phenotype to alleviate human bias in interpreting these data.

Supplemental Tables

Supplemental Table 3. siRNAs targeting *EBF2.* Related to Supplemental Figure 2

	Cat No-Primer No	Primer name	Sequence
siEBF2 #1	10620318-437913 A09	Ebf2MSS203753	(RNA)-CCA UAG CCU AUG AGG GAC AGA AUA A
	10620319-437913 A06	Ebf2MSS203753	(RNA)-UUA UUC UGU CCC UCA UAG GCU AUG G
siEBF2 #2	10620318-445249 B02	Ebf2MSS203754	(RNA)-CCC UCU ACG GCA CAC CAC ACA AUA A
	10620319-445249 A10	Ebf2MSS203754	(RNA)-UUA UUG UGU GGU GUG CCG UAG AGG G

Supplemental Table 4. shRNA clones against *Ebf2*. Related to Figure 1 and Figure 6

**Name**	Target sequence
shRNA Clone 1	GCAGCAATTCTTTGTTATGAA
shRNA Clone 2	GCCTTAAATGAACCCACCATA
shRNA Clone 3	CGGCTACAGCAATGTTCCTAT
shRNA Clone 4	GTTCATCTACACAGCCTTAAA
shRNA Clone 5	GACAGAATAAGAATCCCGAAA

Supplemental Table 7. Sequences of Taqman primers used for RT-qPCR. Related to Figure 2, Figure 6, and Supplemental Figures 2, 3, 5, 6, and 8

**Gene**	**Ref Seq Number**	**Probe Sequence**	**Primer Sequence**
Adipoq	NM_009605	5’-/56-FAM/ACGACACCA/ZEN/AAAGGGCTCAGGAT/3IABkFQ/-3’	5’-TGTCTGTACGATTGTCAGTGG-3’<br>5’-GCAGGATTAAGAGGAACAGGAG-3’
Pparg	NM_011146	5`-/56-FAM/CCACCAACT/ZEN/TCGGAATCAGCTCTGT /3IABkFQ/-3’	5’CTGATGCACTGCCTATGAGC-3’<br>5’-AAGGAATGCGAGTGGTCTTC-3’
Fabp4	NM_024406	5`-/56-FAM/TGAAGAGCA/ZEN/TCATAACCCTAGATGGCG/3IABkFQ/-3’	5’-AAATCACCGCAGACGACAG-3'<br>5’-CCTTTCATAACACATTCCACCAC-3’
Rplp0	NM_007475	5`-/56-FAM/AGGCCCTGC/ZEN/ACTCTCGCTT/3IABkFQ/-3'	5’-TTATAACCCTGAAGTGCTCGAC-3’<br>5’-CGCTTGTACCCATTGATGATG-3'
Ucp1	NM_009463	5’-/56-FAM/AAACACCTG/ZEN/CCTCTCTCGGAAACAA/3IABkFQ/-3’	5’-CAAATCAGCTTTGCCTCACTC-3’<br>5’-CACACCTCCAGTCATTAAGCC-3’
Cidea	NM-007702	5’-/56-FAM/AGACATCCA/ZEN/GAGTCTTGCTGATAAGTTCCT/3IABkFQ /-3’	5’-TCAAACCATGACCGAAGTAGC -3’<br>5’-GTAACCAGGCCAGTTGTGAT -3’

Supplemental Table 8. Sequences of SYBR Green primers used for RT-qPCR. Related to Figure 2, Figure 6, and Supplemental Figures 2, 3, 4, and 5

Gene	Ref Seq Number	Primer Sequence
Lep	NM_008493	5'-GTGCCTATCCAGAAAGTCCAG -3'<br>5'-AATGAAGTCCAAGCCAGTGA-3'
Rplp0	NM_007475	5'-TTATAACCCTGAAGTGCTCGAC-3'<br>5'-CGCTTGTACCCATTGATGATG-3'
Lpl	NM_008509	5'-TCTAACTGCCACTTCAACCAC-3'<br>5'-GTCAGGTTCTCTCTTGTACAGG-3'
Ebf2 exon 2-3	NM_010095	5'-GAGATGGATTCGGTCAGGTC-3'<br>5'-CAAGTTTGAAGGAGGTTGCTTT-3'
Ebf1	NM_007897	5'-TCGTACAAGTCCAAGCAGTTC-3'<br>5'-GGAATGACCTTCTGTAACCTCTG-3'
Ebf3	NM_010096	5'-CCCTCTCCTACAAGTCTAAGCA-3'<br>5'-TGTCTGGGTATCACTTTCTGC-3'
Col1a1	NM_007742	5'-CGCAAAGAGTCTACATGTCTAGG-3'<br>5'-CATTGTGTATGCAGCTGACTTC-3'

Supplemental Table 9. Key resources table

REAGENT or RESOURCE	SOURCE	IDENTIFIER
Antibodies		
Rabbit Anti-Mouse Perilipin Polyclonal Antibody, Unconjugated	Cell Signaling Technology	Cat# 3470, RRID:AB_2167268
Rabbit Anti-Collagen Type I	Rockland	Cat#600-401-103-0.1, RRID:AB_2074625
Rabbit Anti-Collagen Type VI	Rockland	Cat# 600-401-108-0.1, RRID:AB_217576
Rabbit Anti-CD34	Cell signaling	Cat#26233S
UCP1 (C-17)	Santa Cruz Biotechnology	Cat# sc-6528, RRID:AB_2304265
Fibronectin (EP5)	Santa Cruz Biotechnology	Cat#sc-8422, RRID:AB_627598
F(ab')2-Goat anti-Rabbit IgG (H+L) Cross-Adsorbed Secondary Antibody, Alexa Fluor™ 594	ThermoFisher Scientific	Cat#A11072, RRID:AB_2534116
Donkey anti-Goat IgG (H+L) Cross-Adsorbed Secondary Antibody, Alexa Fluor™ 647	ThermoFisher Scientific	Cat# A-21447, RRID:AB_2535864
Biological samples		
Patient-derived white adipose tissue samples	University of Michigan, Department of Surgery, Ann Arbor, Michigan	N/A
Patient-derived liver samples	University of Michigan, Department of Pathology, Ann Arbor, Michigan	N/A
Patient-derived kidney samples	University of Michigan, Department of Pathology, Ann Arbor, Michigan	N/A
Healthy adult woman's white adipose tissue samples	University of Michigan, Department of Surgery, Ann Arbor, Michigan	N/A
Chemicals, peptides, and recombinant proteins		
Dulbecco's modified Eagle's medium (DMEM)	ThermoFisher Scientific	Cat#11965-092
Fetal bovine serum (FBS)	ThermoFisher Scientific	Cat#A3160402
Penicillin-Streptomycin (10,000 U/mL)	ThermoFisher Scientific	Cat#15140122
Dexamethasone	Sigma-Aldrich	Cat#D1756

Porcine insulin	Sigma-Aldrich	Cat#I5523
3-Isobutyl-1-methylxanthine (IBMX)	Sigma-Aldrich	Cat#I5879
Rosiglitazone	Cayman Chemical	Cat# 71740
Triiodo-L-thyronine (T3)	Sigma-Aldrich	Cat#T2877
PBS (1X), pH 7.4	ThermoFisher Scientific	Cat#10010-023
Collagenase, Type 2	Worthington	Cat# LS004176
Collagenase D	Sigma-Aldrich	Cart#11088866001
Polybrene	Sigma-Aldrich	Cat#TR-1003-G
Lipofectamine RNAiMAX Transfection Reagent	ThermoFisher Scientific	Cat#13778030
Applied Biosystems TaqMan Universal PCR Master Mix	ThermoFisher Scientific	Cat#4304437
Applied Biosystems SYBR Green PCR Master Mix	ThermoFisher Scientific	Cat#4309155
Triton X-100	Sigma-Aldrich	N/A
BODIPY 493/503	ThermoFisher Scientific	Cat#D3922
DAPI	ThermoFisher Scientific	Cat#D3571
Puromycin	Sigma-Aldrich	#CatP7255
Critical commercial assays		
NimbleGen SeqCap EZ kit v3.0	Roche	N/A
HiSeq X Ten Reagent Kit v2.5	Illumina	N/A
Insulin rat/mouse insulin ELISA kit	Millipore Sigma	Cat#EZRMI-13K
Mouse Adipokine Magnetic Bead Panel	Millipore Sigma	Cat#MADKMAG-71K
Mouse Adiponectin Single Plex Magnetic Bead Panel	Millipore Sigma	Cat#MADPNMAG-70K
Glucose Hexokinase assay kit	Randox Laboratories	Cat#GL8319
Triglycerides (GPO-PAP) assay kit	Randox Laboratories	Cat# TR8332
QIAshredder	Qiagen	Cat#79654
RNeasy Mini Kit	Qiagen	Cat#74104
iScript cDNA Synthesis Kit 100 Rxn	Bio-Rad	Cat#1708891
Custom Taqman assay, mEbf2_c493G_T	ThermoFisher Scientific	Assay ID: ANPR7KU,
Dual-Luciferase Reporter Assay	Promega	Cat#PROE1910
Deposited data		
Mouse RNA-Seq data	This paper	Gene Expression Omnibus (GEO) database: GSE288829
Human RNA-seq data	This paper	Gene Expression Omnibus (GEO) database: GSE288824
Raw data for graphs	This paper	Supporting Data Values file
Experimental models: Cell lines		
3T3-L1	ATCC	Cat#CL-173
Human subcutaneous preadipocytes	Cell Applications	Cat#802s-05a
Adipose tissue Stem cell fraction (SVF)	This paper	N/A
HEK293	ATCC	Cat# CRL-1573
Cos-7 cells	ATCC	Cat# CRL-1651

Experimental models: Organisms/strains		
Mouse: C57BL/J	Jackson Laboratory	#000664
Mouse: *Ebf2* $^{E165X/+}$	This paper	N/A
Oligonucleotides		
sgRNA C359A targets the 5' region of the exon 7 of *Ebf2 for CRISPR/Cas9*	5' TTGGGGTCCCGAGGCTACCT PAM (AGG) 3'	N/A
sgRNA C359Y targets the 3' region of the exon 7 of *Ebf2 for* CRISPR/Cas9	5' CAGCCAGGATTTCCTCATGT PAM (TGG) 3'	N/A
Amplicon C359Y forward primer for Sanger sequences	5' TGGTCTATTTTCTACATTCTCGC ACCTTT 3'	N/A
Amplicon C359A reverse primer for Sanger sequences	5': AGGAAGGAAAGAAACTGAAGAG CAGAAAT 3'	N/A
Amplicon C359Y forward primer for Sanger sequences	5' CCAGCTTCAGATTGAGGGTTTG AAAAATA 3'	N/A
Amplicon C359Y reverse primer for Sanger sequences	5': CCACAAAACACGGTTTTTAACAA TGTCAC 3'	N/A
siRNA oligonucleotides targeting EBF2	Supplemental Table 3	N/A
shRNA oligonucleotide sequences and targets for human and mouse *EBF2* gene	Supplemental Table 4	N/A
Forward primer sequence for quantification of *Ebf2* allele-specific transcripts	CATCTTGACTCCTTCCCGTTCTA C	N/A
Reverse primer sequence for quantification of Ebf2 allele-specific transcripts	CGATGATGACCGGGTCTGAAG	N/A
Probe 1 sequence for quantification of Ebf2 allele-specific transcripts	TCGATGCTGTGAGAAGA	N/A
Probe 2 sequence for quantification of Ebf2 allele-specific transcripts	AGTCGATGCTGTTAGAAGA	N/A
Taqman primers sequences for qRT-PCR	Supplemental Table 7	N/A
SYBR Green primers sequences for qRT-PCR	Supplemental Table 8	N/A
Multimerized EBF response element (EBF-RE)	5′-ATTCCCAAGGGAAT gggt ATTCCCAAGGGAAT gggt ATTCCCAAGGGAAT gggt ATTCCCAAGGGAAT gggt ATTCCCAAGGGAAT gggt ATTCCCAAGGGAAT-3′	N/A
Multimerized ZNF423 response element (ZNF423-RE)	5′-CCGCCC gggt CCGCCC gggt CCGCCC gggt CCGCCC gggt CCGCCC gggt CCGCCC-3′	N/A
Multimerized ZNF521 response element (ZNF521-RE	5′-GACCACCCA gggt GACCACCCA gggt GACCACCCA	N/A

	gggt GACCACCCA gggt GACCACCCA-3′	
Recombinant DNA		
pLL-RSV-EBF2-WT-CMVeGFP	This paper	NA
pLL-RSV-EBF2-E165-stop-CMVeGFP	This paper	N/A
pLL-RSV-EBF1-CMV-eGFP	This paper	N/A
pLKO-EBF2-81513	This paper	N/A
pLKO-EBF2-81514	This paper	N/A
pLKO-EBF2-81515	This paper	N/A
pLKO-EBF2-81516	This paper	N/A
pLKO-EBF2-81517	This paper	N/A
pGL3-4XEBF2 enhancer SV40 Luc	This paper	N/A
pGL3-EBF-RE	This paper	N/A
pGL3-ZNF423-RE	This paper	N/A
pGL3-ZNF521-RE	This paper	N/A
pGL3-SV40Luc	Promega	N/A
Software and algorithms		
ImageJ (for image analysis)	NIH	https://imagej.nih.gov/ij/
Prism 10	GraphPad Software	https://www.graphpad.com/scientific-software/prism/
Adobe Illustrator	Adobe	https://www.adobe.com/products/illustrator.html
GATK HaplotypeCaller version 4.0	Genome Analysis Toolkit (GATK)	N/A
TapeStation analysis software v3.2	Agilent Technologies	N/A
IPathway	Advaita Corporation	
Other		
Standard rodent chow diet (5L0D)	LabDiet	N/A
High Fat Diet (45% kcal as fat, 4.7 kcal/g)	Research Diets	Cat#D12451
Olympus IX71fluorescent microscope	Olympus	N/A
Transmission electron microscope (TEM; JEM 1400 Plus).	JOEL	N/A

Supplemental Figures

Supplemental Figure 1.

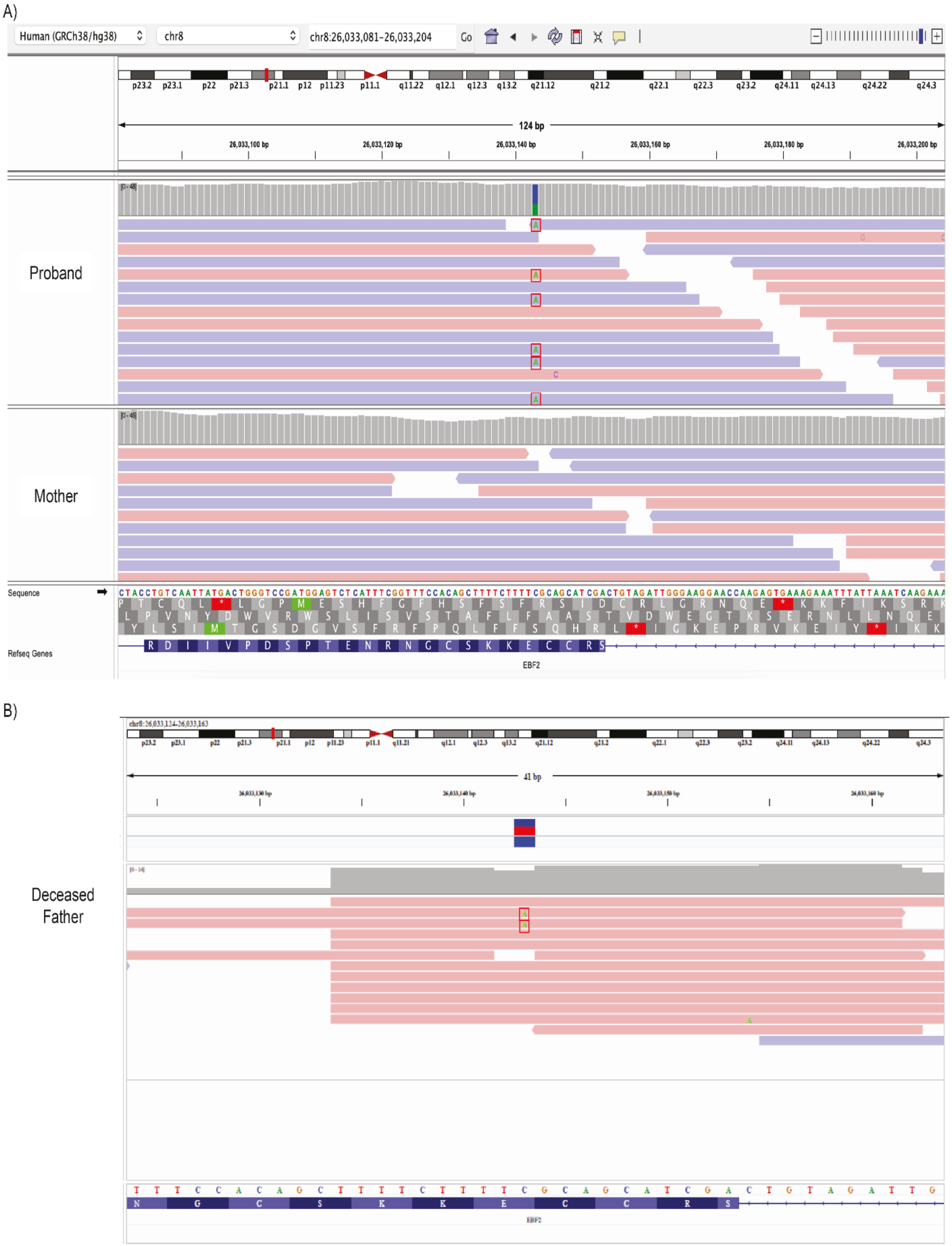

Supplemental Figure 1. Whole genome sequencing confirms inheritance of the EBF2 variant
(A) Whole genome sequencing (WGS) reads from the proband, and her mother at chromosome 8:26033143 show a heterozygous nonsense variant in EBF2 (c.493G>T; p.E165X) in the proband but not in her mother. (B) WGS reads from the proband's father, obtained from postmortem DNA, demonstrate the same variant, confirming paternal inheritance.

Supplemental Figure 2.

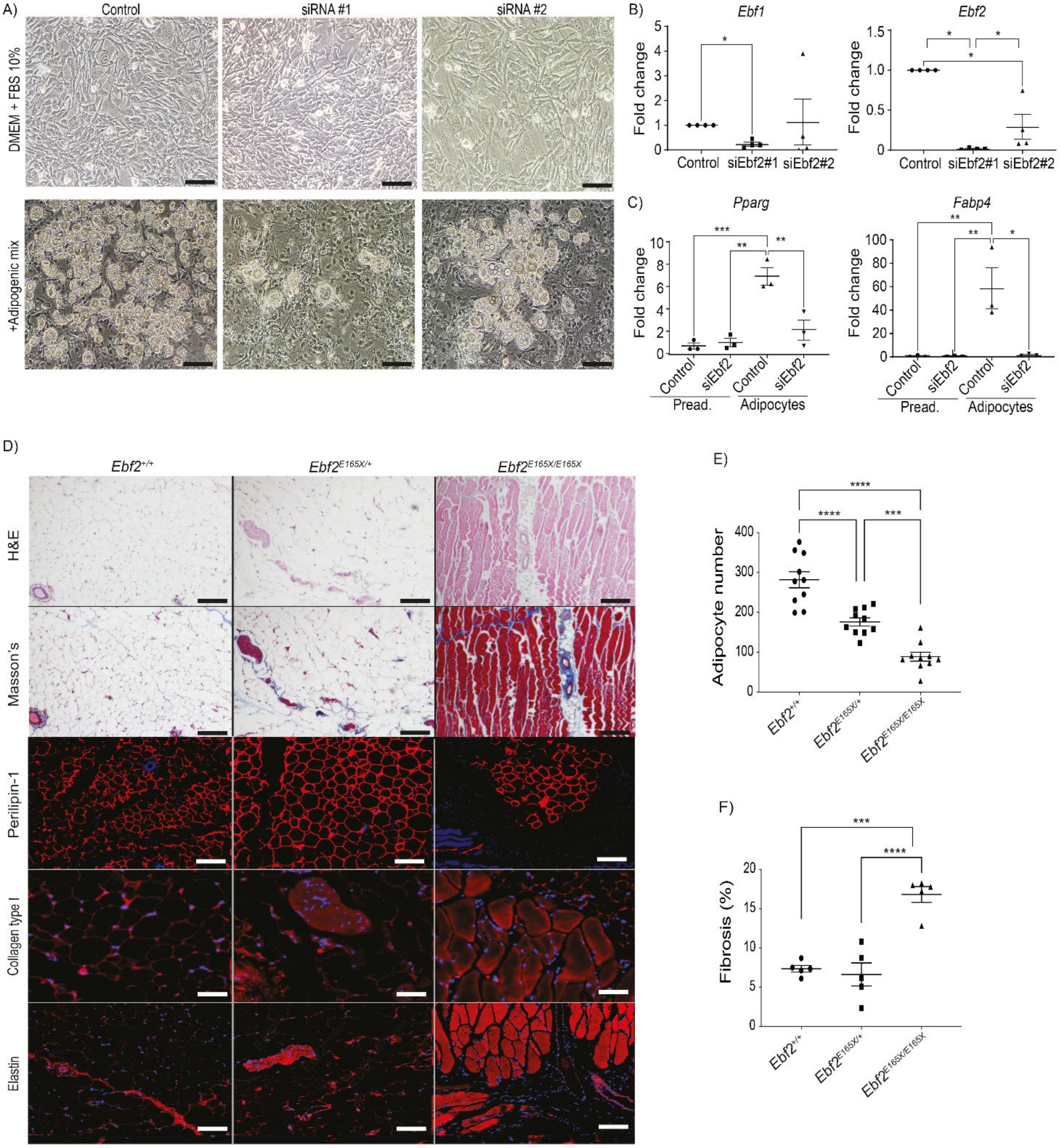

Supplemental Figure 2. *EBF2* p.E165X variant impairs adipogenesis *in vitro* and adipose tissue remodeling *in vivo*

(A) Two independent siRNA clones against *Ebf2* suppressed adipogenesis (siRNA#1 on the middle panels and siRNA#2 on the right panels). (B) *Ebf1* (left) and *Ebf2* (right) expression in both siRNA-transfected cells (n=4). (C) *Pparg* (left) and *Fabp4* (right) expression assessed in adipocytes differentiated after siEbf2#2 treatment (n=3). (D) Hematoxylin and eosin (HE), Masson Trichrome (Masson's) and immunofluorescence staining for perilipin, collagen-1 and elastin in IWAT of $Ebf2^{+/+}$, $Ebf2^{E165X/+}$ and $Ebf2^{E165X/\,E165X}$ mice. (E) Quantification of adipocyte number from IWAT of the indicated mice. Ten randomly chosen HPFs from a single section were analyzed for each mouse (n=1). (F) Histological quantification of IWAT fibrosis (%) in the indicated mice, using the trichrome C positive signal measurement by ImageJ software. Five randomly chosen HPFs from a single section were analyzed for each mouse (n=1). Scale bar, 100 μm (A and D). Two-way ANOVA with Tukey's multiple comparisons test, *p < 0.05, **p < 0.01, ***p < 0.001, ****p < 0.001 (B, C, E and F.) Bar graphs represent mean ± SEM. Each dot represents a biological replication (B and C) or a single HPF (E and F). N = number of biological replications (B and C) or number of animals (E and F). Related to Figure 1 and Figure 6.

Supplemental Figure 3.

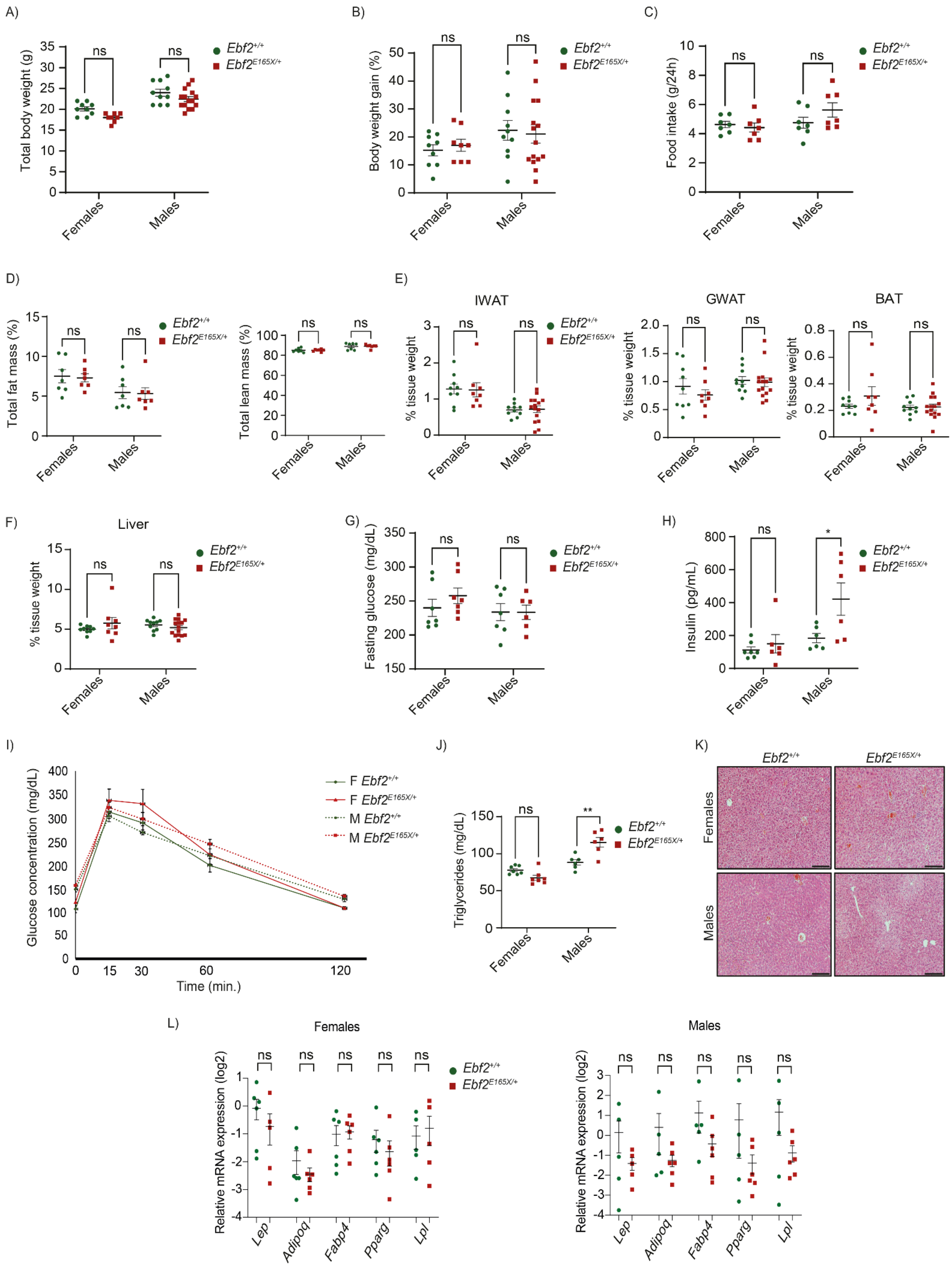

Supplemental Figure 3. Metabolic phenotypes of *Ebf2*$^{+/+}$ and *Ebf2*$^{E165X/+}$ mice fed a chow diet (CD)
(A and B) Total body weight of the indicated 8-week-old fed CD mice (n=9 *Ebf2*$^{+/+}$ and 8 *Ebf2*$^{E165X/+}$ of females; 10 *Ebf2*$^{+/+}$ and 15 *Ebf2*$^{E165X/+}$ of males) (A) and % body weight gain after an additional 8 weeks of CD in the same mice (B).
(C) Daily food intake at 22°C from Comprehensive Laboratory Animal Monitoring System (CLAMS) data of the indicated mice in week 16 of CD (n=7 per group).
(D) Nuclear magnetic resonance (NMR) measurement of the indicated mice's fat and lean mass percentage in week 16 of CD (n=7 per group).
(E and F) IWAT, GWAT, BAT (E), and liver (F) weight (g) normalized to total body weight (g) and expressed as a percentage of the sixteen-week-old indicated mice on CD (n=8-15 per group).
(G and H) Analysis of serum glucose (G) and insulin (H) in sixteen-week-old mice after a 5-hour fast (n=6-7 per group).
(I) Change in plasma glucose levels during the intraperitoneal glucose tolerance test in the indicated mice after 16 weeks of CD (n=9 *Ebf2*$^{+/+}$ and 8 *Ebf2*$^{E165X/+}$ of females; 10 *Ebf2*$^{+/+}$ and 15 *Ebf2*$^{E165X/+}$ of males).
(J) Analysis of serum triglyceride levels in sixteen-week-old mice after a 5-hour fast (n=6-7 per group).
(K) Representative images of H&E-stained liver from sixteen-week-old mice fed CD are shown.
(L) RT-qPCR of *Lep*, *Adipoq*, *Fabp4*, *Pparg,* and *Lpl* genes from GWAT depots of the sixteen-week-old indicated mice on CD (n=5-6 per group). Gene expression was normalized to a housekeeping gene (*Rplp0*). Scale bar, 100 μm (K). Student's t-test (two-tailed, unpaired), ns, not significant (I and L). Two-way ANOVA with Tukey's multiple comparisons test, $*p < 0.05$, $**p < 0.01$, ns, not significant (A, B, C, D, E, F, G, H, and J). Each dot represents a single mouse (A, B, C, D, E, F, G, H, J, and L). Bar graphs represent mean ± SEM. N = number of animals. Related to Figure 2.

Supplemental Figure 4.

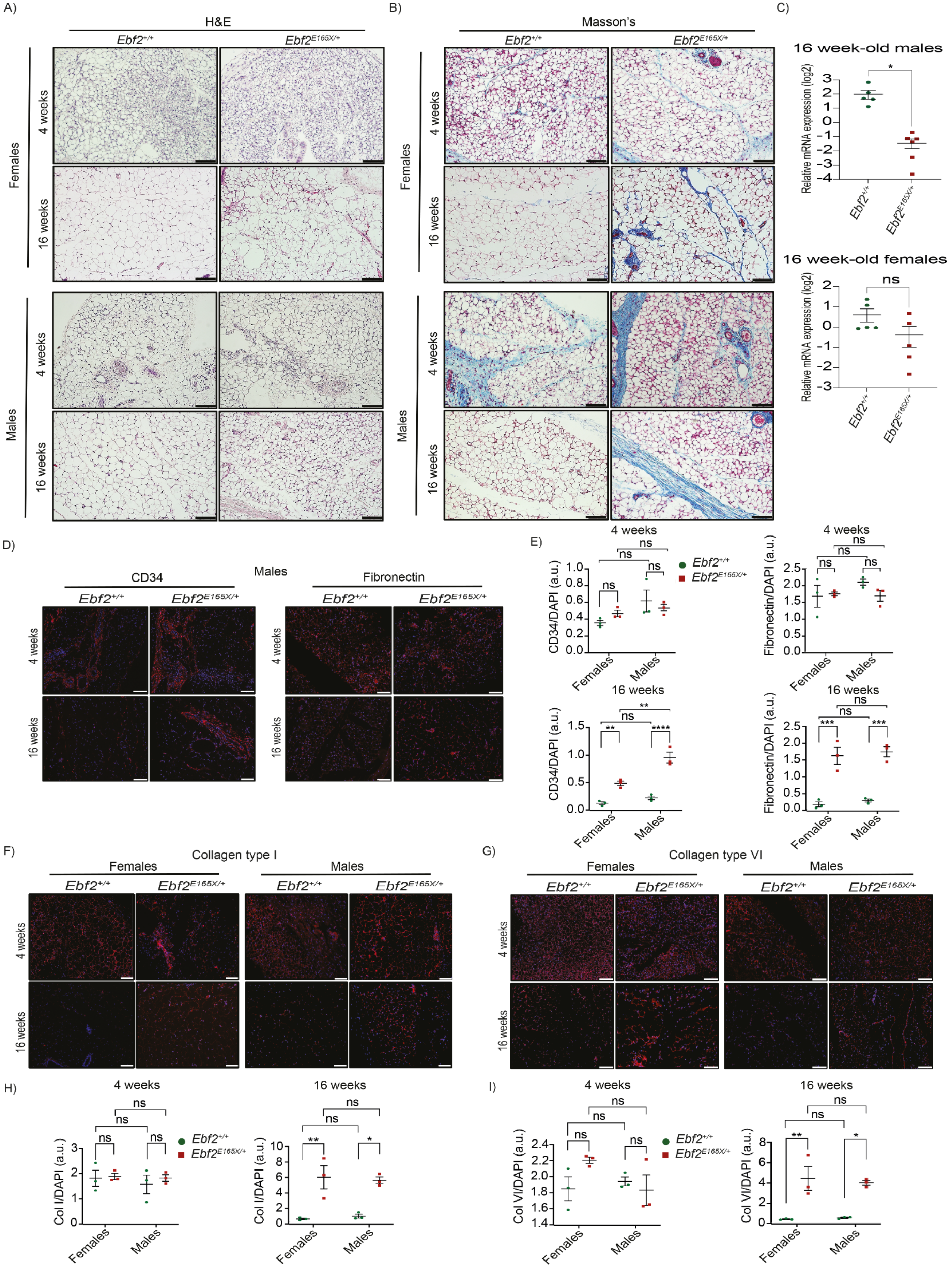

Supplemental Figure 4. Developmental changes of IWAT structure and ECM composition in *Ebf2*$^{+/+}$ and *Ebf2*$^{E165X/+}$ mice fed CD

(A and B) H&E (A) and Masson's trichrome (B) staining of representative IWAT sections of the 4-week-old and 16-week-old indicated mice on CD. Excess collagen deposition is shown in blue in Masson's trichrome staining. Four randomly chosen HPFs from a single section were analyzed for each mouse (n=4). The images from the same 16-week-old mice (male *Ebf2* $^{+/+}$ and *Ebf2* $^{E165X/+}$ and female *Ebf2* $^{E165X/+}$ H&E panels) were used in Figure 2A. These redundant images are included to enable the direct comparison with the younger mice under the same staining and microscopy settings.

(C) RT-qPCR of *Col1a1* gene from IWAT depots of the sixteen-week-old indicated mice on CD (n=5-6 per group). Gene expression was normalized to a housekeeping gene (*Rplp0*).

(D) Immunofluorescence staining for CD34 and fibronectin of the indicated mice on CD. CD34 and fibronectin are shown in red, and nuclei are counterstained with DAPI (blue).

(E) Quantification of immunofluorescence staining for CD34 and fibronectin in IWAT of the indicated mice fed CD (n=3). Data are expressed as CD34/DAPI and fibronectin/DAPI ratios. Top left: CD34/DAPI ratio at 4 weeks; top right: fibronectin/DAPI ratio at 4 weeks; bottom left: CD34/DAPI ratio at 16 weeks; bottom right: fibronectin/DAPI ratio at 16 weeks. Data are expressed as arbitrary units (a.u.).

(F) Immunofluorescence staining for collagen type I of the indicated mice on CD. Collagen I is shown in red, and nuclei are counterstained with DAPI (blue).

(G) Immunofluorescence staining for collagen type VI of the indicated mice on CD. collagen VI is shown in red, and nuclei are counterstained with DAPI (blue).

(H) Quantification of immunofluorescence staining for collagen type I in IWAT of the indicated mice fed CD (n=3). Data are expressed as collagen type I/DAPI ratios. Left panel: col I/DAPI ratio at 4 weeks; right panel: col I/DAPI ratio at 16 weeks. Data are expressed as arbitrary units (a.u.).

(I) Quantification of immunofluorescence staining for collagen type VI in IWAT of the indicated mice fed CD (n=3). Data are expressed as collagen type VI/DAPI ratios. Left panel: Col VI/DAPI ratio at 4 weeks; right panel: Col VI/DAPI ratio at 16 weeks. Data are expressed as arbitrary units (a.u.).

Scale=100 μm (A, B, D, F, and G). Student's t-test (two-tailed, unpaired), $**p < 0.01$, ns, not significant (C). Two-way ANOVA with Tukey's multiple comparisons test, $*p < 0.05$, $**p < 0.01$, $***p < 0.001$, $****p < 0.001$. ns, not significant (E, H, and I). Each dot represents a single mouse (C, E, H, and I). Bar graphs represent mean ± SEM. N = number of animals. Related to Figure 2.

Supplemental Figure 5.

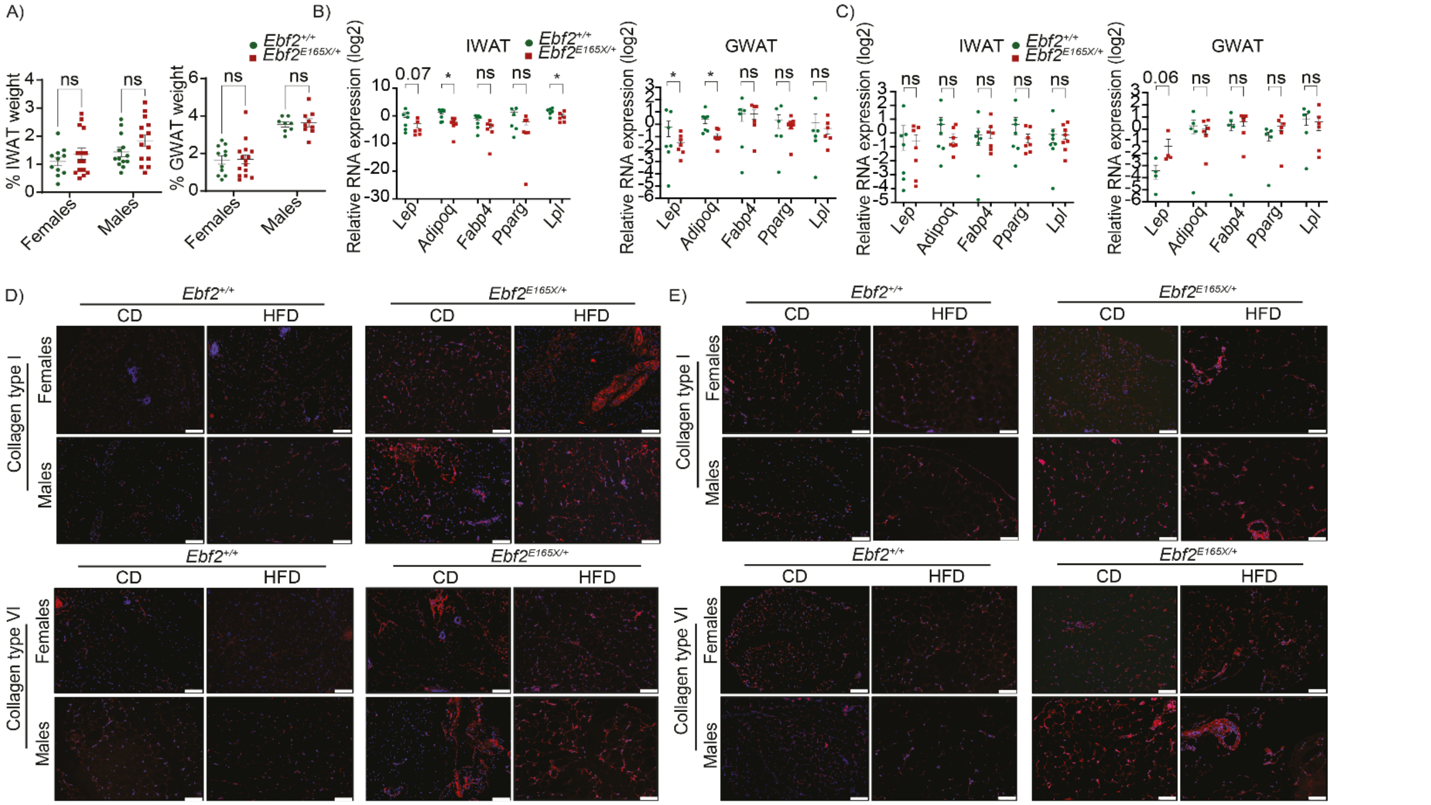

Supplemental Figure 5. White adipose tissue weight and gene expression in *Ebf2*$^{+/+}$ and *Ebf2*$^{E165X/+}$ mice fed high-fat diet (HFD)

(A) IWAT (left) and GWAT (right) weight (g) normalized to total body weight (g) and expressed as percentage of the sixteen-week-old indicated mice at week 8 of HFD (n=8-15 per group).

(B and C) RT qPCR of *Lep*, *Adipoq*, *Fabp4*, *Pparg* and *Lpl* genes from IWAT (upper panels) and GWAT (lower panels) depots of the sixteen-week-old male (B) and female (C) indicated mice at week 8 of HFD (n=4-8 per group). Gene expression was normalized to a housekeeping gene (*Rplp0*).

(D and E) Immunofluorescence staining for collagen type I and collagen type VI in IWAT (D) and GWAT (E) of the indicated sixteen-week-old mice after 2 months of CD or HFD. Collagen type I and collagen type VI are shown in red, and nuclei are counterstained with DAPI (blue).

Scale bar, 100 µm (D and E). Two-way ANOVA with Tukey's multiple comparisons test, ns, not significant (A). Student's t-test (two-tailed, unpaired), *p < 0.05, ns, not significant (B and C). Each dot represents a single mouse (A, B, and C). Bar graphs represent mean ± SEM. N = number of animals. Related to Figures 3 and 4.

Supplemental Figure 6.

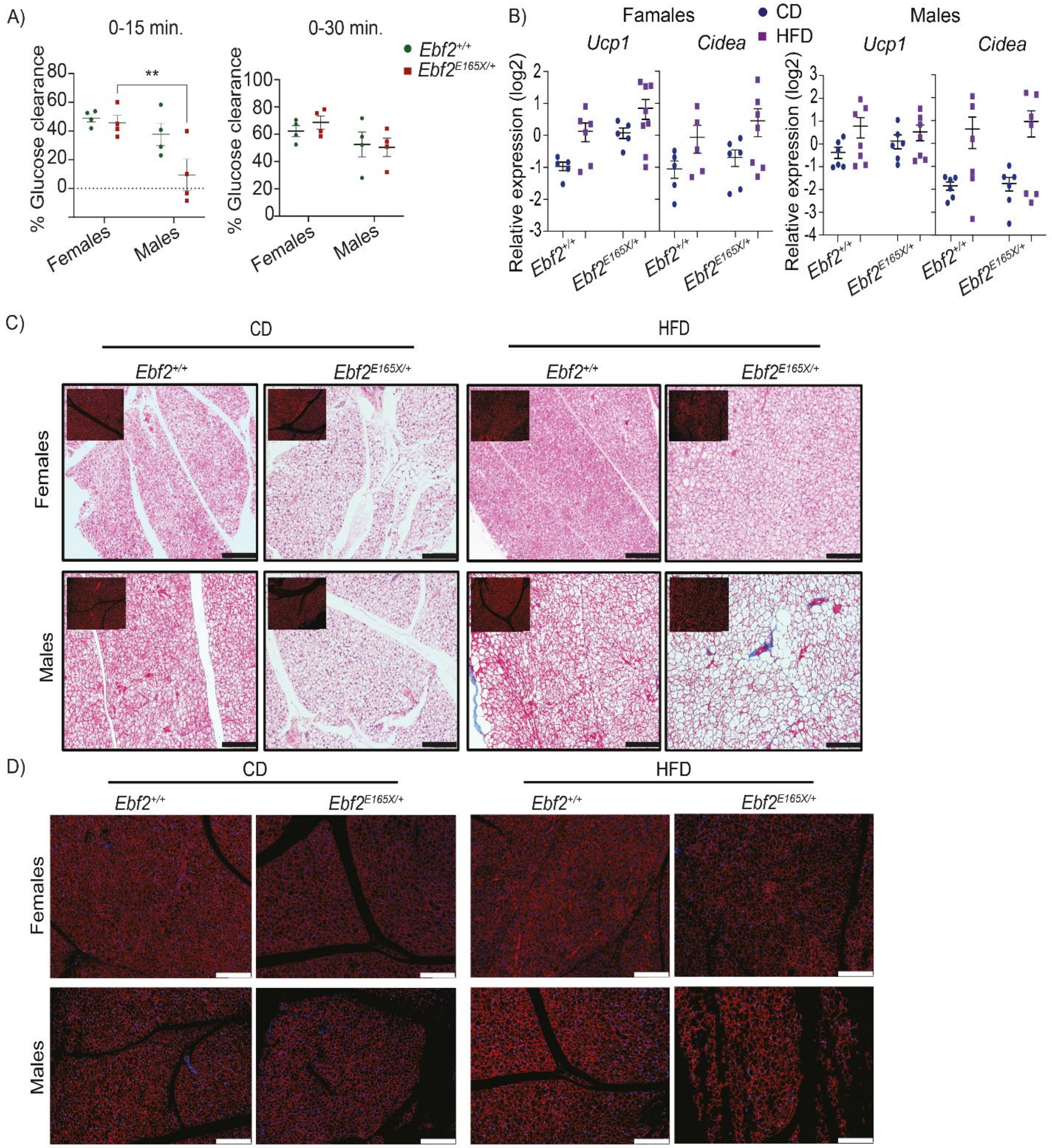

Supplemental Figure 6. *EBF2* p.E165X variant does not impair BAT identity in vivo

(A) Insulin tolerance tests were performed after 8 weeks of HFD feeding in 8-week-old mice (n=4 per group). Following a 4-hour fast, mice received an intraperitoneal injection of insulin (0.75 U/kg). Glucose clearance is presented as a percent change from baseline at 15 and 30 minutes.

(B) *Ucp1* and *Cidea* expression in BAT depots of the sixteen-week-old indicated mice (n=5-8 per group). Gene expression was normalized to a housekeeping gene (*Rplp0*).

(C) Representative images of BAT from the sixteen-week-old indicated mice on CD (left) and HFD (right), stained with H&E and immunofluorescence staining of perilipin (insets). Perilipin is shown in red, and nuclei are counterstained with DAPI (blue).

(D) UCP1 immunofluorescence staining of BAT from the sixteen-week-old indicated mice on CD (left) and HFD (right). UCP1 is shown in red, and nuclei are counterstained with DAPI (blue).

Scale bar, 100 µm (C and D). Two-way ANOVA with Tukey's multiple comparisons tests (A, B). Each dot represents a single mouse (A, B). Bar graphs represent mean ± SEM. N = number of animals. Related to Figures 3 and 4.

Supplemental Figure 7.

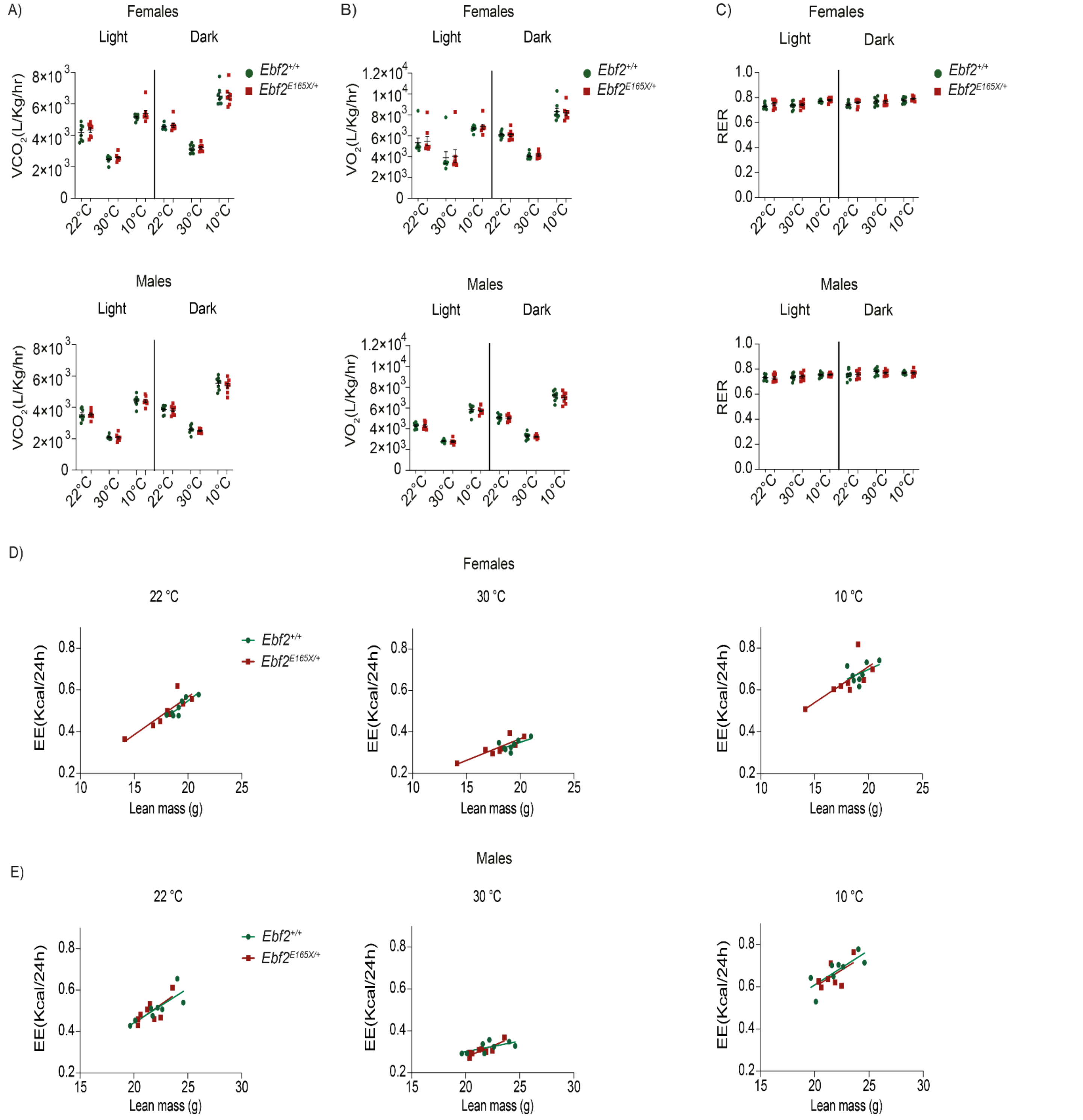

Supplemental Figure 7. Energy expenditure study in *Ebf2*$^{+/+}$ and *Ebf2*$^{E165X/+}$ mice after HFD

(A-C) Data from CLAMS shows VCO2 (A), VO2 (B), and respiratory exchange ratio (RER) (C) of the sixteen-week-old indicated mice in week 8 of HFD, in light and dark, at different temperatures (n=8 per group). (D-E) Linear regression analysis of energy expenditure (EE, kcal/24h) as a function of lean mass in female (D) and male (E) of the sixteen-week-old indicated mice in week 8 of HFD at different temperatures (n=8 per group). Student's t-test (two-tailed, unpaired), ns, not significant (A, B, and C). Each dot represents a single mouse (A, B and C). Bar graphs represent mean ± SEM. N = number of animals. Related to Figures 3 and 4.

Supplemental Figure 8.

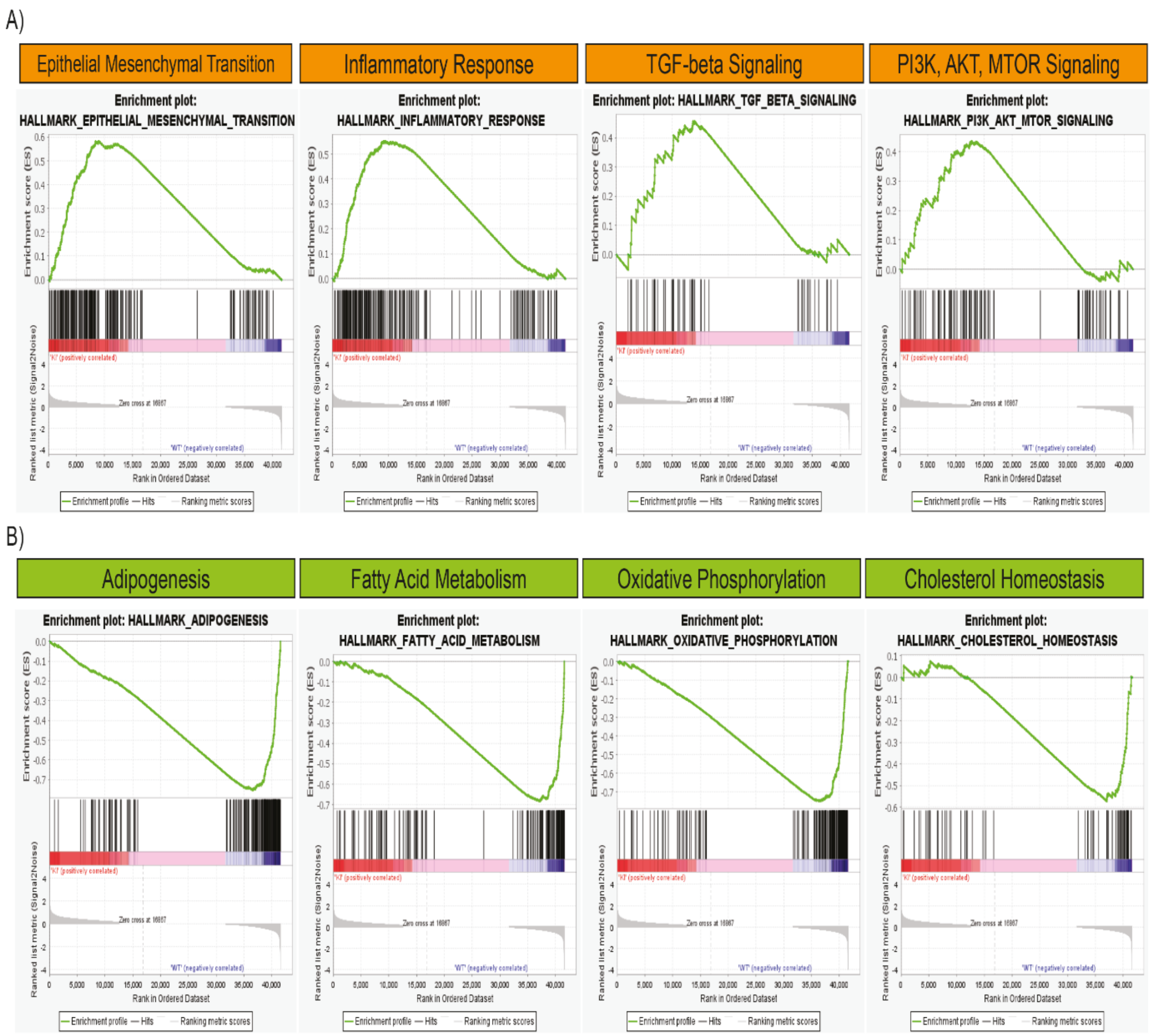

Supplemental Figure 8. Upregulated TGF-beta and inflammatory pathways with concurrent downregulation of mitochondria and fatty acid metabolism pathways in *Ebf2*$^{E165X/+}$ IWAT
Bulk RNA-seq data from IWAT of WT and KI mice were analyzed using GSEA to identify pathways differentially regulated between genotypes. Enrichment plots illustrate representative hallmark pathways significantly upregulated (A) or downregulated (B) in *Ebf2*$^{E165X/+}$ IWAT samples. The normalized enrichment score (NES) and ranked metrics (signal-to-noise) are shown. Heatmaps show the leading-edge gene subsets driving enrichment.

Supplemental Figure 9.

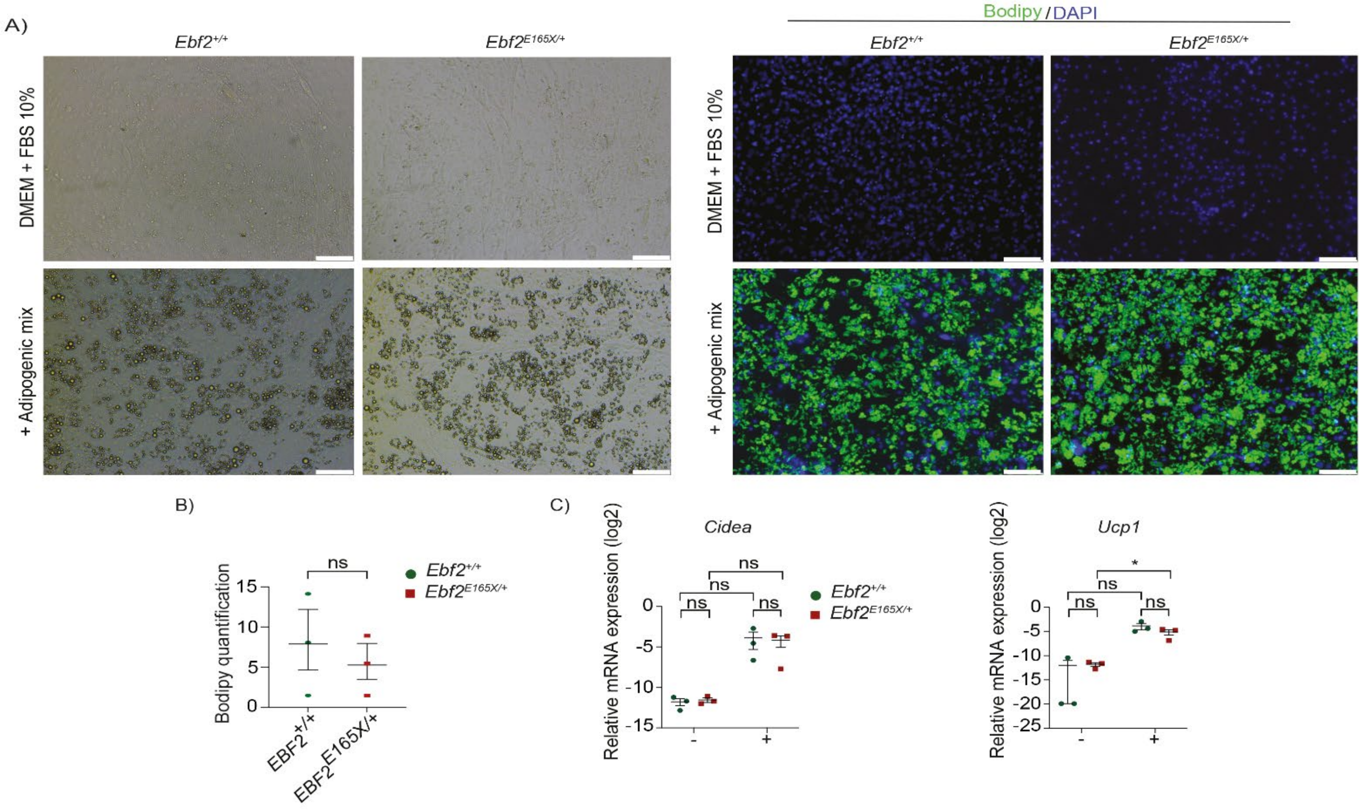

Supplemental Figure 9. The *EBF2* variant does not impair brown adipocyte differentiation in vitro
(A) Adipocyte differentiation of SVF isolated from *Ebf2*$^{+/+}$ and *Ebf2*$^{E165X/+}$ BAT. Bright field on the left and fluorescence imaging on the right. Lipid droplets are shown in green (BODIPI), and nuclei in blue (DAPI) in the right panels.
(B) BODIPY staining quantification in SVF isolated from *Ebf2*$^{+/+}$ and *Ebf2*$^{E165X/+}$ BAT (n=3)
(C) *Cidea and Ucp1* expression normalized to *Rplp0* in *Ebf2*$^{+/+}$ and *Ebf2*$^{E165X/+}$ BAT-derived SVF before (-) and after (+) differentiation (n=3).
Scale bar, 100 µm (A). Student's t-test (two-tailed, unpaired); ns, not significant (B). Two-way ANOVA with Tukey's multiple comparisons tests, ns, not significant (C). Bar graphs represent mean ± SEM. Each dot represents a biological replication (B and C). N = number of biological replications (B and C). Related to Figure 6.

Supplemental Figure 10.

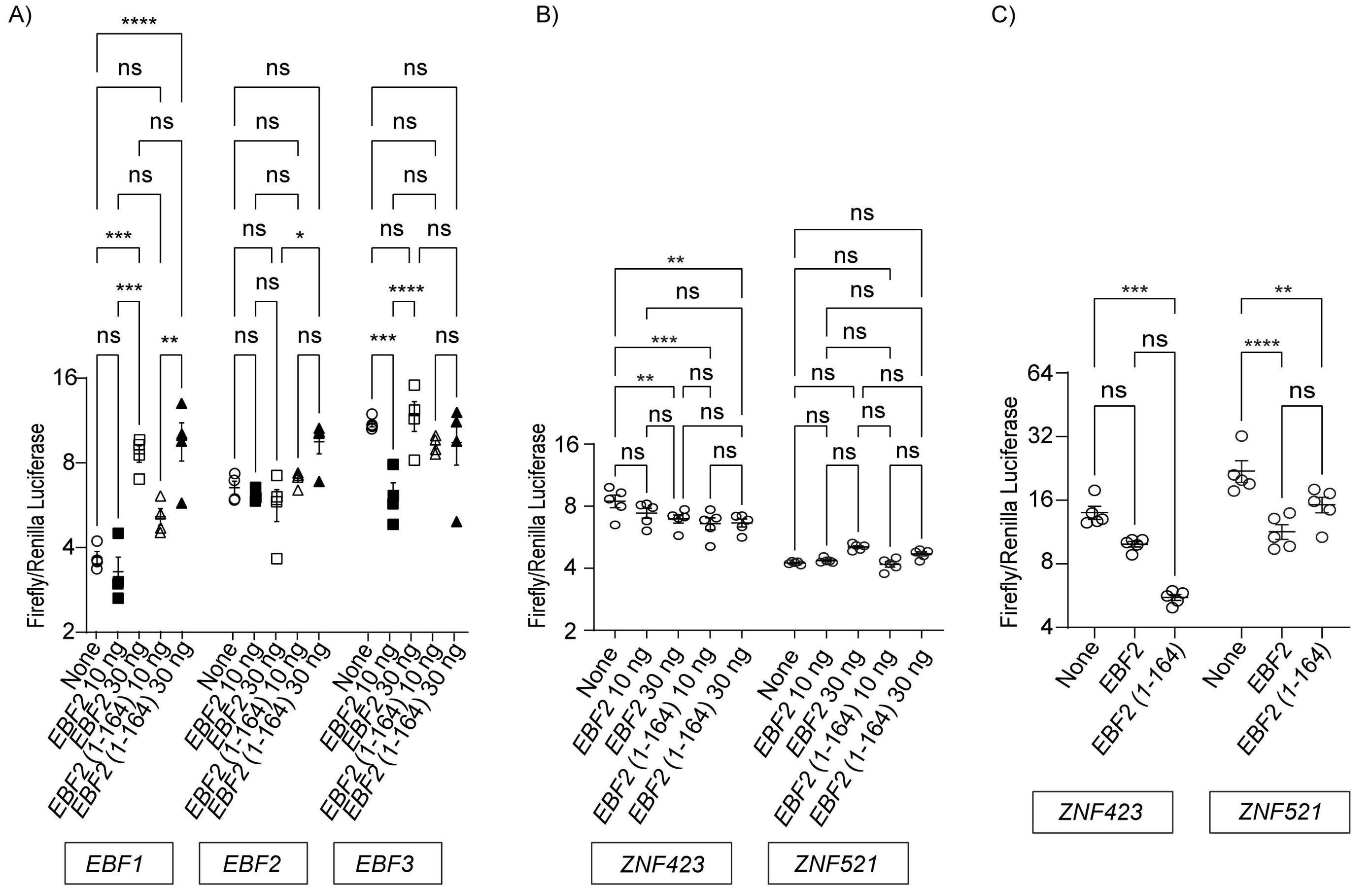

Supplemental Figure 10. Effects of truncated EBF2 (1–164) on EBF- and ZNF-driven synthetic reporter activity.

(A) COS-7 cells were transfected with pGL3-EBF-consensus–luciferase (pGL3-EBF-RE-luc), pRL-SV40, and driver constructs expressing *EBF1*, *EBF2*, or *EBF3*. Full-length *EBF2* or truncated *EBF2* (1–164) was co-transfected at the indicated doses, and luciferase activity was measured.

(B) COS-7 cells were transfected with pGL3-ZNF423–RE-luc or pGL3-ZNF521–RE-luc together with pRL-SV40 and co-transfected with full-length *EBF2* or truncated *EBF2* (1–164) at the indicated doses.

(C) COS-7 cells were transfected with pGL3-EBF-RE-luc, pRL-SV40, and ZNF423 or ZNF521, and co-transfected with 30 ng full-length *EBF2* or truncated *EBF2* (1–164).

For all experiments (A–C), firefly luciferase activity was normalized to Renilla luciferase (n = 5 biological replicates). Data are presented as mean ± SEM. Two-way ANOVA was used to determine statistical significance with Tukey's multiple-comparison test (*p < 0.05, **p < 0.01, ***p < 0.005, ****p < 0.001; ns, not significant). Each dot represents an independent biological replicate.

Supplemental Figure 11.

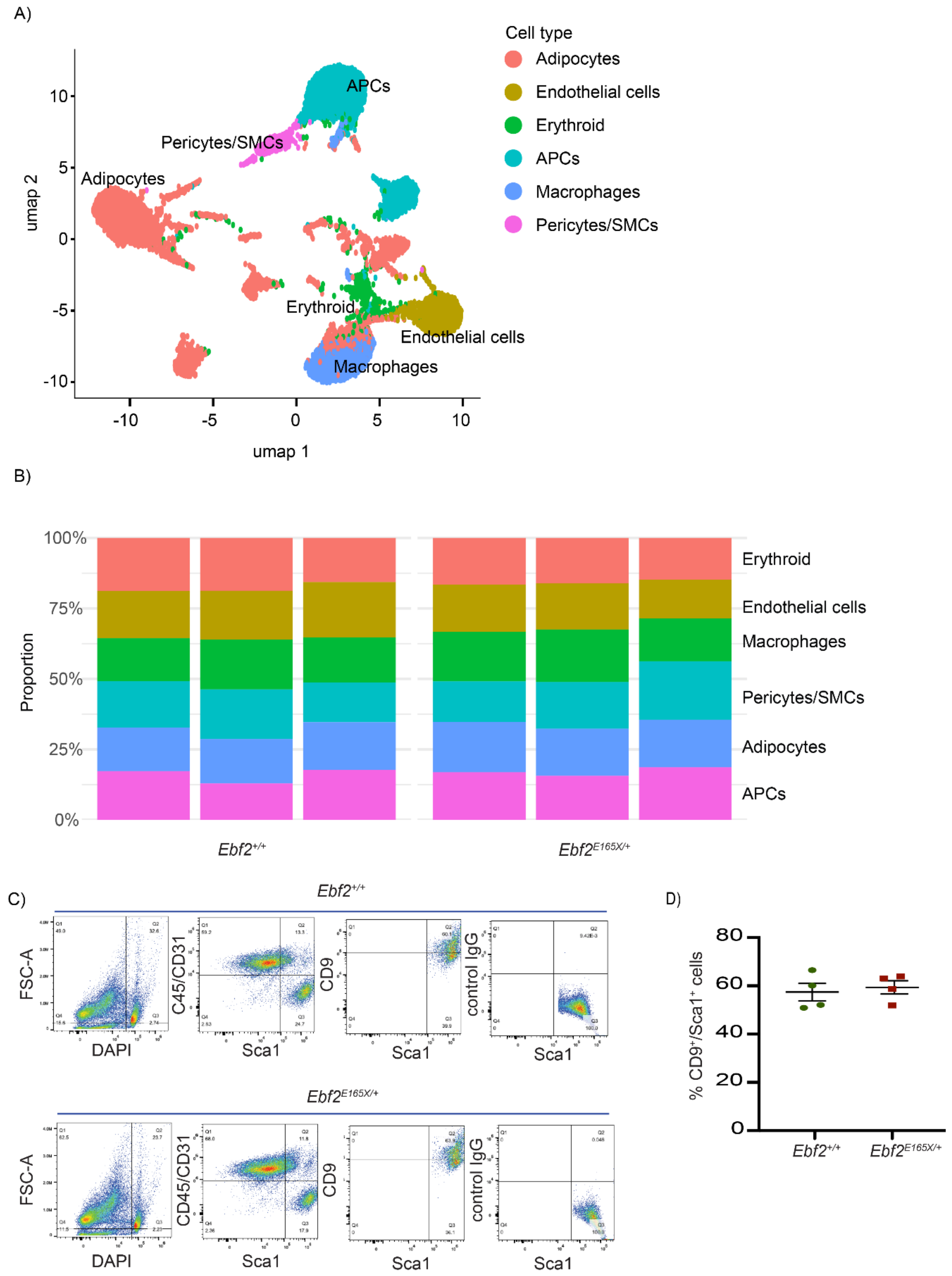

Supplemental Figure 11. Cell type composition and Cd9$^+$ adipocyte precursor proportion in IWAT

(A, B) Cell type deconvolution analysis of bulk RNA-seq data from *Ebf2*$^{+/+}$ and *Ebf2*$^{E165X/+}$ IWAT (n=3) (GSE288829) using a reference snRNA-seq data (GSE288829). UMAP (A) and cell composition heatmap (B).

(C) Flow cytometry analysis of live cells (DAPI negative) to assess the proportion of CD45$^-$CD31$^-$Sca1$^+$Cd9$^+$ IWAT-derived SVFs (n=4 four-week-old mice per group).

(D) %Cd9$^+$ cells per Cd45$^-$Cd31$^-$Sca1$^+$ SVFs (n=4 per group).

REFERENCES

1. Collins RL, Brand H, Karczewski KJ, Zhao X, Alföldi J, Francioli LC, et al. A structural variation reference for medical and population genetics. *Nature.* 2020;581(7809):444-51.
2. Laricchia KM, Lake NJ, Watts NA, Shand M, Haessly A, Gauthier L, et al. Mitochondrial DNA variation across 56,434 individuals in gnomAD. *Genome Res.* 2022;32(3):569-82.
3. Basu S, Xie X, Uhler JP, Hedberg-Oldfors C, Milenkovic D, Baris OR, et al. Accurate mapping of mitochondrial DNA deletions and duplications using deep sequencing. *PLoS Genet.* 2020;16(12):e1009242.
4. Dolzhenko E, Deshpande V, Schlesinger F, Krusche P, Petrovski R, Chen S, et al. ExpansionHunter: a sequence-graph-based tool to analyze variation in short tandem repeat regions. *Bioinformatics.* 2019;35(22):4754-6.
5. Pais LS, Snow H, Weisburd B, Zhang S, Baxter SM, DiTroia S, et al. seqr: A web-based analysis and collaboration tool for rare disease genomics. *Hum Mutat.* 2022;43(6):698-707.
6. Corradi A, Croci L, Broccoli V, Zecchini S, Previtali S, Wurst W, et al. Hypogonadotropic hypogonadism and peripheral neuropathy in Ebf2-null mice. *Development.* 2003;130(2):401-10.
7. Shapira SN, Lim HW, Rajakumari S, Sakers AP, Ishibashi J, Harms MJ, et al. EBF2 transcriptionally regulates brown adipogenesis via the histone reader DPF3 and the BAF chromatin remodeling complex. *Genes Dev.* 2017;31(7):660-73.
8. Haeussler M, Schönig K, Eckert H, Eschstruth A, Mianné J, Renaud JB, et al. Evaluation of off-target and on-target scoring algorithms and integration into the guide RNA selection tool CRISPOR. *Genome Biol.* 2016;17(1):148.
9. Basila M, Kelley ML, and Smith AVB. Minimal 2'-O-methyl phosphorothioate linkage modification pattern of synthetic guide RNAs for increased stability and efficient CRISPR-Cas9 gene editing avoiding cellular toxicity. *PLoS One.* 2017;12(11):e0188593.
10. Hendel A, Bak RO, Clark JT, Kennedy AB, Ryan DE, Roy S, et al. Chemically modified guide RNAs enhance CRISPR-Cas genome editing in human primary cells. *Nat Biotechnol.* 2015;33(9):985-9.
11. Slaymaker IM, Gao L, Zetsche B, Scott DA, Yan WX, and Zhang F. Rationally engineered Cas9 nucleases with improved specificity. *Science.* 2016;351(6268):84-8.
12. Sakurai T, Watanabe S, Kamiyoshi A, Sato M, and Shindo T. A single blastocyst assay optimized for detecting CRISPR/Cas9 system-induced indel mutations in mice. *BMC Biotechnol.* 2014;14:69.
13. Brinkman EK, Chen T, Amendola M, and van Steensel B. Easy quantitative assessment of genome editing by sequence trace decomposition. *Nucleic Acids Res.* 2014;42(22):e168.
14. Moreno-Mateos MA, Vejnar CE, Beaudoin JD, Fernandez JP, Mis EK, Khokha MK, et al. CRISPRscan: designing highly efficient sgRNAs for CRISPR-Cas9 targeting in vivo. *Nat Methods.* 2015;12(10):982-8.
15. Van Keuren ML, Gavrilina GB, Filipiak WE, Zeidler MG, and Saunders TL. Generating transgenic mice from bacterial artificial chromosomes: transgenesis efficiency, integration and expression outcomes. *Transgenic Res.* 2009;18(5):769-85.
16. Allison DB, Paultre F, Maggio C, Mezzitis N, and Pi-Sunyer FX. The use of areas under curves in diabetes research. *Diabetes Care.* 1995;18(2):245-50.
17. Joe AW, Yi L, Natarajan A, Le Grand F, So L, Wang J, et al. Muscle injury activates resident fibro/adipogenic progenitors that facilitate myogenesis. *Nat Cell Biol.* 2010;12(2):153-63.
18. Travis A, Hagman J, Hwang L, and Grosschedl R. Purification of early-B-cell factor and characterization of its DNA-binding specificity. *Molecular and Cellular Biology.* 1993;13(6):3392-400.
19. Tsai RYL, and Reed RR. Identification of DNA Recognition Sequences and Protein Interaction Domains of the Multiple-Zn-Finger Protein Roaz. *Molecular and Cellular Biology.* 1998;18(11):6447-56.
20. Scicchitano S, Giordano M, Lucchino V, Montalcini Y, Chiarella E, Aloisio A, et al. The stem cell-associated transcription co-factor, ZNF521, interacts with GLI1 and GLI2 and enhances the activity of the Sonic hedgehog pathway. *Cell Death Dis.* 2019;10(10):715.
21. Love MI, Huber W, and Anders S. Moderated estimation of fold change and dispersion for RNA-seq data with DESeq2. *Genome Biol.* 2014;15(12):550.
22. Mi H, Muruganujan A, Ebert D, Huang X, and Thomas PD. PANTHER version 14: more genomes, a new PANTHER GO-slim and improvements in enrichment analysis tools. *Nucleic Acids Res.* 2019;47(D1):D419-D26.
23. Draghici S, Khatri P, Tarca AL, Amin K, Done A, Voichita C, et al. A systems biology approach for pathway level analysis. *Genome Res.* 2007;17(10):1537-45.
